# The Analysis of Plasma Proteomics for Luminal A Breast Cancer

**DOI:** 10.1002/cam4.70470

**Published:** 2024-12-06

**Authors:** Meimei Zhao, YongWei Jiang, Xiaomu Kong, Yi Liu, Peng Gao, Mo Li, Haoyan Zhu, Guoxiong Deng, Ziyi Feng, Yongtong Cao, Liang Ma

**Affiliations:** ^1^ Department of Clinical Laboratory China‐Japan Friendship Hospital Beijing China

**Keywords:** biomarker, early detection, luminal A breast cancer, plasma proteomics, prognosis

## Abstract

**Background:**

Breast cancer is the prevailing malignancy among women, exhibiting a discernible escalation in incidence within our nation; hormone receptor‐positive (HR+) human epidermal growth factor receptor 2‐negative (HER2−) breast cancer is the most common subtype. In this study, we aimed to search for a non‐invasive, specific, blood‐based biomarker for the early detection of luminal A breast cancer through proteomic studies.

**Methods:**

To explore new potential plasma biomarkers, we applied data‐independent acquisition (DIA), a technique combining liquid chromatography and tandem mass spectrometry, to quantify breast cancer‐associated plasma protein abundance from a small number of plasma samples in 10 patients with luminal A breast cancer, 10 patients with benign breast tumors, and 10 healthy controls.

**Results:**

The proteomes of 30 participants in all cohorts were analyzed using the DIA method, and a total of 517 proteins and 3584 peptides were quantified. We found that there were significant differences in plasma protein expression profiles between breast cancer patients and non‐breast cancer patients, and breast cancer was mainly related to lipid metabolism pathways. Finally, the optimal protein combinations for the diagnosis of breast cancer were PON3, IGLV3‐10, and IGHV3‐73 through multi‐model analysis, which had a high prediction accuracy for breast cancer (AUC = 0.92), and the model could also distinguish breast cancer from HC (AUC = 0.92) and breast cancer from benign breast tumor (AUC = 0.91).

**Conclusions:**

The study revealed proteomic signatures of patients with luminal A breast cancer, identified multiple differential proteins, and identified three plasma proteins as potential diagnostic biomarkers for breast cancer. It provides a reference for the screening of biomarkers for breast cancer.

## 1 Introduction

Breast cancer is widely recognized as the predominant malignant tumor affecting women in clinical settings. In the year 2020, breast cancer emerged as the leading cause of new cancer cases worldwide. According to the World Health Organization, a staggering 2.3 million women received a breast cancer diagnosis, resulting in 685,000 fatalities globally [1]. Moreover, breast cancer ranks highest among malignancies affecting women in China, with approximately 420,000 new cases reported in 2020, accounting for 19.9% of all reported cases. Notably, the incidence of breast cancer exhibits a trend toward younger age groups, and the prognosis varies significantly depending on the stage of diagnosis. The 5‐year relative survival rates for patients diagnosed with breast cancer between 2012 and 2018 were found to be greater than 99% for stage I, 93% for stage II, 75% for stage III, and 29% for stage IV [2]. Consequently, enhancing the rate of early breast cancer detection and providing prompt and efficacious treatment are imperative for enhancing the survival outcomes of individuals with breast cancer [3].

Currently, guidelines recommend that the most commonly used diagnostic tools for screening and preventing breast cancer include color doppler ultrasonography, X‐rays, mammograms, and breast magnetic resonance imaging [2]. The limitations of ultrasound and magnetic examination are that the detection rate and sensitivity of early microlesions are low, and they cannot reliably distinguish between benign and malignant tumors [3, 4]. X‐ray examination and mammogram send radiation to the human body; Chinese women have less breast fat and more glands and connective tissue, which can interfere with the mammogram and affect the final diagnosis, which may lead to some early patients missing the test [5, 6]. Breast tissue biopsy is the gold standard for diagnosing breast cancer, but biopsy is invasive, which limits its use in population screening. So far, several potential blood‐based biomarkers for breast cancer screening and prevention have been reported, including CEA, CA125, CA153, and so forth, but they all lack sensitivity and specificity as independent markers [7, 8]. Therefore, there is an urgent need for non‐invasive, simple, repeatable, and cost‐effective screening strategies to improve the early detection of breast cancer.

With the rapid development of proteomic techniques based on mass spectrometry (MS), the application of proteomic techniques to find new tumor biomarkers has become a hot topic in tumor research. Proteomics technology has been successfully used in the screening of multiple tumor biomarkers such as colon cancer and lung cancer [9, 10, 11], and since proteins are the main performers of cell function and are the direct drug targets of most current tumor treatments, high‐dimensional proteomic data may provide unprecedented insights to help new biomarker identification and clinical implementation. Proteomics data‐independent acquisition (DIA) is an MS technique that allows for unbiased acquisition of data. Unlike traditional data‐dependent acquisition (DDA), DIA technology uses a preset ion window for data acquisition in MS, covering all possible ion fragments [12]. This allows specific fragments of the protein of interest to be detected and quantified regardless of whether they are in a preset window. The comprehensiveness and high throughput of this data make proteomics DIA a powerful tool for quantitative analysis and functional studies [13, 14].

Based on the expression of estrogen or progesterone receptor markers and human epidermal growth factor 2 (HER2), breast cancers can be divided into three categories: hormone receptor (HR)‐positive/HER2‐negative, HER2‐positive, and triple‐negative [15]. HR‐positive/HER2‐negative breast cancer is the most common subtype, accounting for 65% to 70% of all breast cancers [16], within this category, luminal A breast cancer exhibits the highest incidence rate. Although the overall prognosis for this type of breast cancer is good, some patients still face a high risk of long‐term recurrence [17, 18]. Therefore, the search for tumor markers with high specificity and sensitivity is of great significance for the early diagnosis and treatment of this type of breast cancer. In this study, proteomic DIA technology was applied to screen the key differential proteins that changed in the serum of patients with luminal A breast cancer, and the information analysis was preliminary experimental verification, which provided a basis for the subsequent research on the molecular mechanism of breast cancer occurrence and development and the search for potential targets for clinical breast cancer diagnosis and treatment.

## 2 Methods

### 2.1 Study Object

The test cohort comprised 10 individuals diagnosed with breast cancer, 10 individuals with benign breast tumors, and 10 healthy women who underwent physical examinations at the medical examination center during the same period. These participants were recruited from the China‐Japan Friendship Hospital between December 2021 and February 2023. All 10 breast cancer patients were diagnosed with breast invasive ductal carcinoma through puncture biopsy, following the AJCC criteria [19]. All participants provided written informed consent, and the study protocol adhered to the principles outlined in the Declaration of Helsinki II and was approved by the hospital's ethics committee.

In the morning, 4 mL of fasting venous blood was collected and subsequently left at a temperature of 4°C for a duration of 1 h. Following this, the blood was centrifuged at a speed of 3500 rpm for a period of 5 min, resulting in the separation of the serum. 200 μL of each serum sample was aliquoted into multiple centrifuge tubes, and the serum was stored in a freezer at −80°C for future use. When performing MS analysis, a centrifuge tube containing 200 μL of serum per sample is transported to dry ice, and all serum samples must be protected from repeated freezing and thawing to ensure consistent and reproducible results. In addition to the aforementioned procedures, demographic and clinical data, including age, sex, body mass index (BMI), menstrual history, and family history, were gathered. The inclusion criteria for this study encompass several factors. (1) participants must fall within the age range of 18 to 79 years; (2) patients with breast cancer must have received confirmation of their diagnosis through histopathology, with no presence of any other malignant tumor; additionally, these patients should not have undergone any surgical procedures, chemotherapy, or radiation therapy prior to blood sample collection; (3) individuals with benign breast tumors must not exhibit any other tumors aside from benign breast disease; (4) control patients should not have any tumors or a history of other breast diseases. Other criteria excluded participants with (1) systemic chronic conditions, such as cardiovascular disease, hypertension, and diabetes; (2) metabolic‐related conditions, such as phenylketonuria; and (3) pregnant and lactating women.

### 2.2 Preparation of Plasma Samples for Proteomics

The sample was combined with the lysis buffer, consisting of 1% SDC, 100 mM Tris–HCl (pH = 8.5), 10 mM TCEP, and 40 mM CAA, and incubated at a temperature of 60°C for a duration of 30 min to achieve complete protein reduction and alkylation. The protein concentration of the resulting supernatant was determined using the Bradford method. To ensure equal protein amounts across different samples, the solution containing 1% SDC and 100 mM Tris–HCl (pH = 8.5) was used to bring all samples to the same volume. The SDC concentration was reduced to below 0.5% by adding an equal volume of ddH2O. Trypsin was then added at a ratio of 1:50 (enzyme: protein, w/w) for overnight digestion at a temperature of 37°C.

### 2.3 LC–MS/MS Analysis

All samples were subjected to analysis using the timsTOF Pro (Bruker Daltonics), a hybrid trapped ion mobility spectrometer (TIMS) quadrupole time‐of‐flight mass spectrometer. The timsTOF Pro was coupled to an UltiMate 3000 RSLCnano system (Thermo) equipped with a CaptiveSpray nano ion source (Bruker Daltonics). Peptide samples were introduced into a C18 Trap column (75 μm × 2 cm, 3 μm particle size, 100 Å pore size, Thermo) and subsequently separated on a reversed‐phase C18 analytical column (75 μm × 25 cm, 1.6 μm particle size, 100 Å pore size, IonOpticks). Mobile phases A and B, consisting of 0.1% formic acid in water and 0.1% formic acid in ACN, respectively, were employed to establish a separation gradient lasting 60 min. This gradient entailed an initial increase from 6% to 11% B within 5 min, followed by a subsequent increase to 25% B within 35 min, and further elevation to 50% B within 15 min. Additionally, a 3‐min washing step at 90% B and a subsequent 2‐min re‐equilibration at 6% B were implemented. The flow rate was maintained at 300 nL/min. The mass spectrometers were operated in diaPASEF mode, with the capillary voltage set at 1400 V. The acquisition of MS and MS/MS spectra was conducted within the m/z range of 100 to 1700. The ion mobility was scanned over a range of 0.6 to 1.6 Vs/cm^2^. Both the accumulation time and ramp time were set at 100 ms. The diaPASEF acquisition scheme was established based on the m/z‐ion mobility plane, utilizing the timsControl software provided by Bruker Daltonics. The collision energy was linearly ramped, correlating with the mobility values, starting at 59 eV when 1/K0 equaled 1.6 Vs/cm^2^ and decreasing to 20 eV when 1/K0 equaled 0.6 Vs/cm^2^.

### 2.4 Proteomics Data Analysis

The DIA raw data were subjected to analysis using DIA‐NN (V1.8.1) in library‐free mode. The spectra files were searched against the reviewed Human protein database obtained from UniProt. Protein intensities were normalized using the MaxLFQ algorithm. The “PG.MaxLFQ” column present in the DIA‐NN output tables was utilized for conducting quantitative analysis at the protein level. The database offers functional annotations of the identified proteins through the utilization of GO (Gene Ontology, www.geneontology.org), KEGG (http://www.genome.jp/kegg/), and DO (Diseases Ontology, https://disease‐ontology.org/). In the processing of the proteomics data, the initial step involved the removal of contaminated proteins, followed by the implementation of logarithmic transformation. Subsequently, missing values were imputed using values that corresponded to a normal distribution centered around the detection limit of the mass spectrometer. In summary, the mean and standard deviation of the distribution of the real intensities were initially calculated. Subsequently, a new distribution was generated by shifting the values by 1.8 standard deviations downward and adjusting the width to 0.25 standard deviations. These derived values were then utilized to impute the missing data, thereby facilitating statistical analysis. Lastly, any protein exhibiting a missing ratio exceeding 50% in both groups was excluded, while the remaining proteins were chosen for further analysis.

### 2.5 Statistical Analysis

The pretreatment of data is subjected to descriptive statistical analysis, wherein continuous variables that exhibit a normal distribution are assessed using means and standard deviations, while those that do not conform to a normal distribution are evaluated using quartiles. Categorical variables are described in terms of proportions or rates. A t‐test is employed for data that adhere to a normal distribution, whereas a rank sum test is utilized for data that do not conform to a normal distribution. The primary screening of biomarkers relies on the selection of fold change and a statistical test with a significance level of *p* ≤ 0.05 as the basis. LASSO regression was employed to conduct additional screening of biomarkers. Subsequently, the model was constructed using the chosen biomarkers in accordance with the prevailing circumstances. Prior to modeling, it is recommended to partition the entire dataset, allocating 70% of the data for training purposes and reserving 30% for testing. The evaluation of the model will be conducted through three distinct perspectives. Firstly, discrimination of the model will be assessed using the C‐Index, area under the curve (AUC), or C‐Statistics. Secondly, the calibration of the clinical prediction model will be evaluated by examining the calibration curve. Lastly, the clinical effect of the model will be assessed through decision curve analysis (DCA) clinical decision curve analysis or nomogram. Statistical analysis and model building methods will be analyzed using R or Python.

## 3 Results

### 3.1 Demographic Attributes of the Individuals Constituting the Test Cohort and the Validation Cohort

A proteomic analysis was conducted on plasma samples to examine variations in protein expression among individuals with breast cancer, benign conditions, and healthy controls (HC), with the aim of identifying potential diagnostic markers for breast cancer patients. The test cohort consisted of 10 breast cancer patients (all women), 10 benign patients (all women), and 10 HC patients (all women). Notably, there were no statistically significant differences observed in age, BMI, CA125 levels, blood glucose levels, aspartate aminotransferase (AST) levels, and creatinine levels across the three groups. However, the tumor marker CA15‐3 exhibited higher levels in the breast cancer group compared to both the benign group and the HC group. The demographic and clinical characteristics of the test and validation groups are presented in Tables 1 and 2, offering a comprehensive overview of these groups.

**TABLE 1:** The demographic attributes of the individuals constituting the test cohort.

Characteristics	BC patients	Benign patients	Healthy control	*p*
Race	Han ethnic	Han ethnic	Han ethnic	—
Age (years, mean ± SD)	43.70 ± 8.37	43.30 ± 13.70	42.10 ± 16.37	0.961
BMI (mean ± SD, kg/m^2^)	22.55 ± 2.36	21.42 ± 2.47	22.53 ± 4.30	0.666
CA153 (mean ± SD, U/mL)	14.19 ± 3.71	8.19 ± 2.57	9.27 ± 4.01	0.001
CA125 (mean ± SD, U/mL)	13.20 ± 8.24	17.94 ± 11.51	15.36 ± 4.65	0.477
Glucose (mean ± SD, mmol/L)	5.51 ± 0.44	5.18 ± 0.72	5.08 ± 0.56	0.237
AST (mean ± SD, U/L)	18.60 ± 5.17	16.40 ± 3.31	17.80 ± 4.82	0.555
SCR (mean ± SD, μmol/L)	54.77 ± 7.18	54.44 ± 4.81	54.64 ± 8.13	0.994

Abbreviations: AST, aspartate aminotransferase; BMI, body mass index; SCR, serum creatinine.

**TABLE 2:** Clinical information of 10 breast cancer patients in this study.

Patient no.	Age	Tumor stage	Lymph node status	Molecular subtype	Luminal subtype			
					ER	PR	HER2	Ki67
P1	40	T1	N0	Luminal A	+	+	−	3%
P2	38	T2	N0	Luminal A	+	+	−	9%
P3	41	T1	N0	Luminal A	+	+	−	6%
P4	33	T2	N1	Luminal A	+	+	−	8%
P5	36	T2	N0	Luminal A	+	+	−	7%
P6	54	T2	N1	Luminal A	+	+	−	10%
P7	41	T1	N1	Luminal A	+	+	−	5%
P8	60	T3	N1	Luminal A	+	+	−	10%
P9	46	T1	N0	Luminal A	+	+	−	11%
P10	48	T2	N0	Luminal A	+	+	−	8%

*Note:* “+” means positive; “−” means negative.

Abbreviations: ER, estrogen receptor; HER2, human epidermal growth factor receptor 2; PR, progesterone receptor.

### 3.2 Plasma Protein Profile of Luminal A Breast Cancer

**FIGURE 1:**
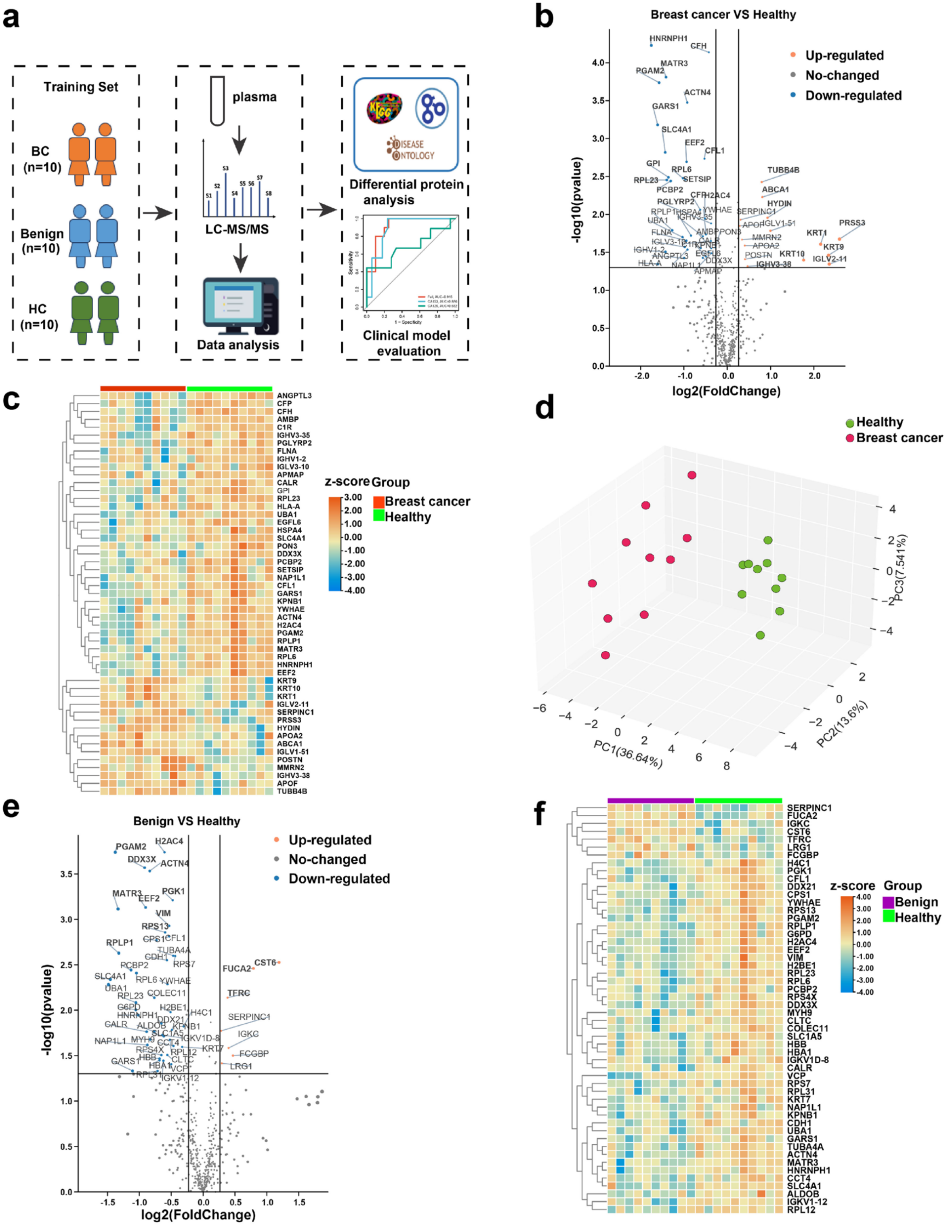
Proteomic analysis revealed distinct variations among breast cancer, benign breast tumors, and control groups. (a) Schematic diagram of the study design; blood samples were collected from 10 patients with luminal A breast cancer, 10 patients with benign breast tumors, and 10 healthy controls. Plasma samples were then analyzed using high‐throughput, MS‐based proteomic techniques. A learning model was established to distinguish between the different populations and evaluate the diagnostic ability of the diagnostic model, which combined clinical indicators and proteomic characteristics. (b) The presented diagram illustrates a volcano map showcasing the differentially expressed proteins observed between the BC group and the HC group. Each data point within the map corresponds to a distinct protein, whereby the color orange signifies an up‐regulated expression, while the color blue signifies a down‐regulated expression. (c) Heat maps of differentially expressed proteins between the BC group and the HC group. Protein expression is subjected to row normalization to obtain Z‐scores, which are subsequently subjected to hierarchical clustering. The correlation coefficient (right) serves as an indicator of the positive or negative correlation exhibited by each protein. Blue: negative correlation; red: positive correlation. (d) Principal component analysis (PCA) plot of the BC group (red) and the HC group (green); each dot represents a sample. (e) Volcanic map of differential expression of proteins in the benign group and HC group. (f) Quantitative heat maps of differentially expressed proteins in the benign group and HC group.

To evaluate specific protein profiles of luminal A breast cancer, a proteomic analysis was conducted on paired plasma samples using LC–MS/MS in a training cohort (Figure 1a). This analysis successfully identified and quantified a total of 517 proteins and 3584 peptides (Table S1); an overview of total sample proteins, peptides, and quality control and analysis of proteomic results is shown in Figure S1. The raw proteomic data were subjected to analysis using the DIA_NN software (version 1.8.1), and the final results were meticulously screened for parent ion and protein levels at a false discovery rate (FDR) of 1%. Then the differences in protein expression profiles were computed for each pairwise comparison, namely, breast cancer versus HC, benign versus HC, and breast cancer versus benign. Subsequently, we employed pairwise comparative volcano maps and heat maps to visually represent the variations in protein expression between breast cancer, benign, and HC.

For the breast cancer group and HC group, proteomic differential abundance analysis of the whole cohort showed that 51 proteins were significantly dysregulated in the breast cancer group (|log2FC| ≥ 0.26, *p* ≤ 0.05) (Table S2). Compared to the HC group, 15 proteins were up‐regulated in the breast cancer group and 36 proteins were down‐regulated in the breast cancer group (Figure 1b). The most statistically distinct differentially expressed proteins (DEPs) in elevated levels included tubulin beta‐4B chain (TUBB4B), phospholipid‐transporting ATPase (ABCA1), and hydrocephalus‐inducing protein (HYDIN), while down‐regulated DEPs consisted of heterogeneous nuclear ribonucleoprotein H (HNRNPH1), complement factor H (CFH), and matrin‐3 (MATR3). Based on quantitative data pertaining to the differential proteins utilized for heat mapping, it was determined that there existed a significant disparity in the expression levels of said proteins between the two aforementioned groups (Figure 1c). The principal component analysis conducted on a set of 51 proteins exhibiting differential expression further substantiated the ability to discern between the breast cancer group and the HC group based on the aforementioned DEPs (Figure 1d).

For the benign group and HC group, 52 proteins were significantly dysregulated in the benign breast tumor group (|log2FC| ≥ 0.26, *p* ≤ 0.05). Compared with the HC group, 7 proteins were up‐regulated in the benign group and 45 proteins were down‐regulated in the benign group. Significantly up‐regulated in benign breast tumors were the proteins cystatin‐M (CST6), fucosidase alpha L2 (FUCA2), transferrin receptor protein 1 (TFRC), immunoglobulin kappa constant (IGKC), leucine‐rich alpha‐2‐glycoprotein (LRG1), IgGFc‐binding protein (FCGBP), and antithrombin‐III (SERPINC1), whereas the proteins histone H2A type 1‐B/E (H2AC4) and phosphoglycerate mutase 2 (PGAM2) were significantly down‐regulated compared to the control group (Figure 1e). Based on quantitative data pertaining to the differential proteins utilized for heat mapping, it was determined that there existed a significant disparity in the expression levels of said proteins between the two aforementioned groups (Figure 1f).

For the breast cancer group and benign group, proteomic differential abundance analysis of the whole cohort showed that 35 proteins were significantly dysregulated in the breast cancer group (|log2FC| ≥ 0.26, *p* ≤ 0.05). Compared to the benign group, 15 proteins were up‐regulated in the breast cancer group and 20 proteins were down‐regulated in the breast cancer group (Figure S2a,b). The principal component analysis performed on a collection of 36 proteins displaying differential expression further supports the capability to distinguish between the breast cancer group and the benign group based on the previously mentioned DEPs (Figure S2c). The findings of this study suggest that there exist discernible variations in protein profiles among individuals with breast cancer and HC and benign and HC, as well as between breast cancer and benign.

### 3.3 Identification of Plasma Protein Signatures in Luminal A Breast Cancer

**FIGURE 2:**
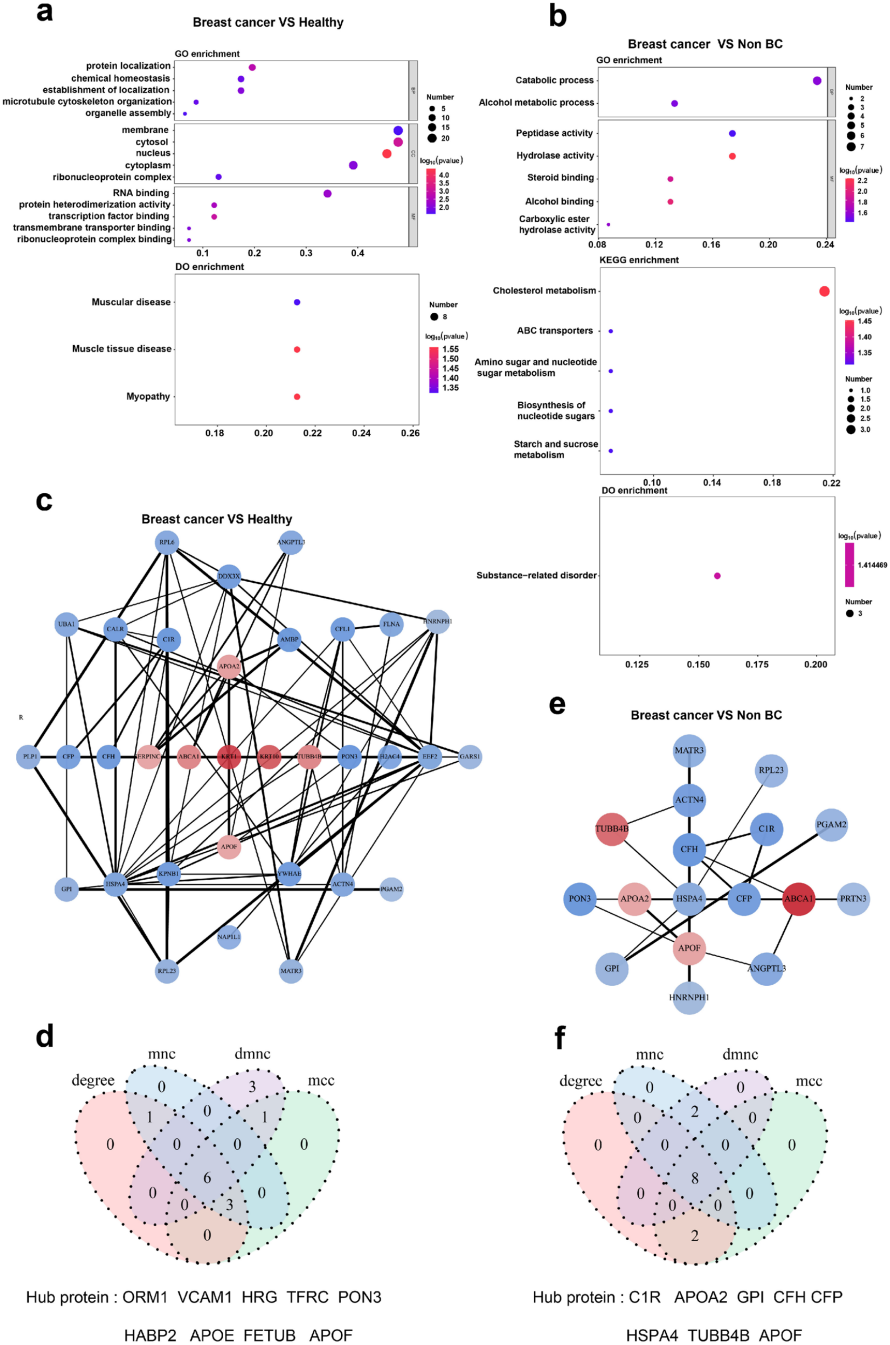
Differential protein analysis in breast cancer and non‐breast cancer. (a) Fifty‐one differentially expressed proteins between BC and HC group were analyzed by DO and GO. DEP enrichment analysis based on GO and DO (two‐tailed hypergeometric test; *p* ≤ 0.05). GO items are sorted by *p*‐value, and the first five items of each category are selected for display. DO entries are sorted by *p*‐value, and the first 15 are selected for display (KEGG is not enriched). (b) GO, DO, and KEGG analysis of differential proteins between BC and non‐breast cancer (benign and HC). GO‐based enrichment analysis of DEPs (two‐sided hypergeometric test; *p* ≤ 0.05). GO terms were sorted by *p*‐value, and the top five terms of each category were displayed; KEGG‐based enrichment analysis of DEPs (two‐sided hypergeometric test; *p* ≤ 0.05). KEGG terms were sorted by *p*‐value, and the top 15 terms were displayed; DO‐based enrichment analysis of DEPs (two‐sided hypergeometric test; *p* ≤ 0.05), DO terms were sorted by *p*‐value, and the top 15 terms were displayed. (c) Protein interaction networks for the diagnosis of relevant proteins between BC and HC group. The dots represent proteins, the lines between the dots represent protein interactions, and the width of the lines represents interaction strength. PPI network diagram of core protein obtained by four algorithms: DEGREE, DMNC, MCC, and MNC. (d) Venn diagram of hub proteins between BC and HC group. (e) PPI network analysis between BC and non‐breast cancer (benign and HC). (f) Venn diagram of hub proteins between BC and non‐breast cancer.

In order to explore potential proteins suitable for early detection of breast cancer, the DEP dataset underwent enrichment analyses utilizing Gene Ontology (GO), Kyoto Encyclopedia of Genes and Genomes (KEGG) pathway, and Disease ontology (DO). Subsequently, R software was employed to conduct functional analyses, encompassing biological processes (BP), molecular functions (MF), and cell components (CC) of the DEPs, based on the Gene Ontology (GO) database. “Protein localization” (GO: 0008104) and “membrane” (GO:0016020) are the most enriched processes of breast cancer (Figure 2a). Differential proteins were assigned annotations to various KEGG signaling pathways; however, upon conducting hypergeometric enrichment analysis, no functional entries were identified as significantly associated with the differential proteins. Consequently, the implementation of bubble mapping was rendered unfeasible. In order to find out the biological function and signaling pathway of the significant enrichment of differential proteins in breast cancer, we analyzed the enrichment of proteins obtained from the breast cancer group and non‐breast cancer group (benign group and HC group) and found that breast cancer is closely related to catabolic process, cholesterol metabolism, and substance‐related disorder (Figure 2b, Table S3). These results highlight that lipid metabolic pathway may play a key role in the pathogenesis of breast cancer.

In this research, only GO terms, KEGG pathways, and DO terms that were statistically enriched (*p* ≤ 0.05) through the hypergeometric test were taken into account. Subsequently, the CytoScape software was employed to construct network diagrams of DEPs utilizing protein–protein interaction (PPI) data obtained from the STRING database (Figure 2c,e). Furthermore, employing the “CytoHubba” plug‐in, the hub protein was determined using four algorithms: DEGREE, DMNC, MCC, and MNC (Table S4). The most significant proteins identified by each algorithm were selected as hub proteins and subsequently cross‐analyzed and mapped onto a Venn diagram (Figure 2d,f).

### 3.4 The Value of Plasma Differential Protein in the Diagnosis of Breast Cancer

**FIGURE 3:**
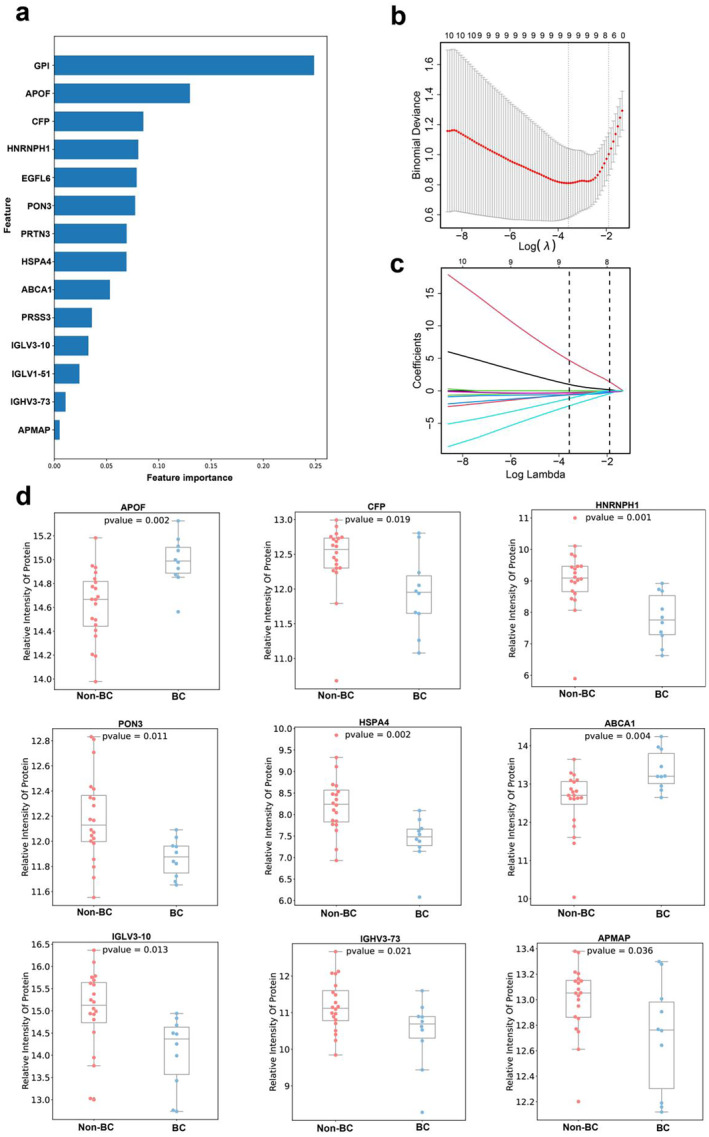
Biomarker inference based on XGBoost machine learning. (a) Importance plot of proteins; 14 important differential proteins were screened in the breast cancer group and the non‐breast cancer group. (b, c) The LASSO regression model performed five‐fold cross‐validation to further screen out nine differential protein features. (d) Relative strengths of nine differentially beneficial protein signatures in breast and non‐breast cancer groups.

**FIGURE 4:**
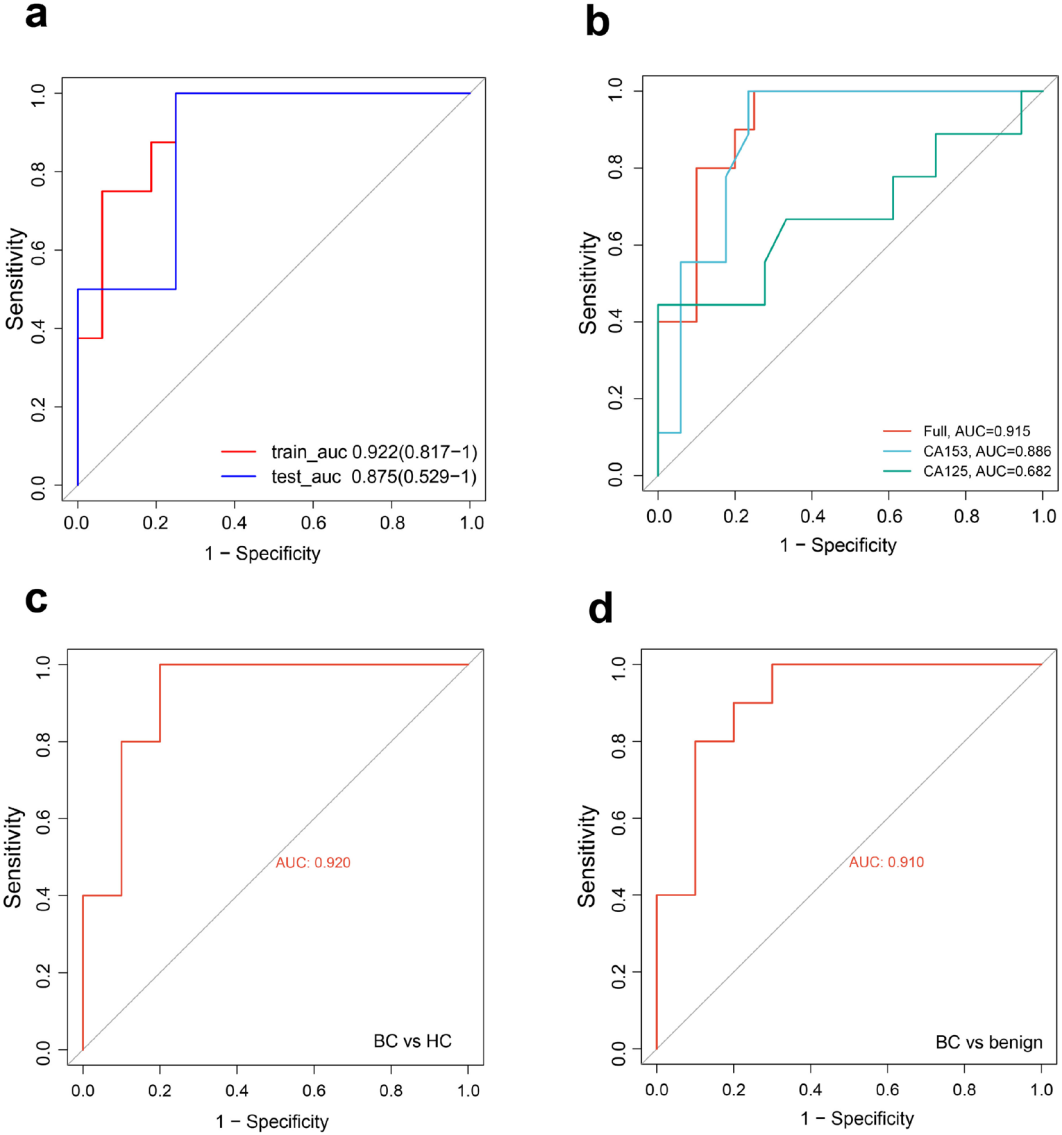
Construction of the breast cancer diagnostic model and evaluation of model diagnostic efficacy. (a) ROC curves in the training and test cohorts for predicting diagnostic performance based on the final screened protein combinations. (b) The ROC curves of all samples (training cohort + test cohort) were used to predict the diagnostic effect based on the final screened protein combinations, and the ROC curves of CA153 and CA125 to predict the diagnostic effect. (c) The diagnostic efficacy of the constructed diagnostic model was verified in BC and HC. (d) The diagnostic efficacy of the constructed diagnostic model was verified in BC and benign.

The primary objective of breast cancer screening is to distinguish individuals with breast cancer from the general population. To achieve this, we conducted an analysis of the distinct proteins found in the breast cancer group compared to the non‐breast cancer group (benign and HC). A total of 30 differential proteins were identified, and the XGBoost machine learning model was employed to identify 14 significant proteins from this set (Figure 3a). Subsequently, the five‐fold cross‐validation LASSO regression model was then used for further screening (Figure 3b,c), in which nine DEP protein signatures were identified (Figure 3d). Then, random combinations of proteins were carried out, three‐fold cross‐validation screening was performed, and the optimal combination was selected by indexes such as AUC value to build a logistic regression model. The optimal variable combination was PON3, IGLV3‐10, and IGHV3‐73. We found that the AUC values for breast cancer diagnosis in the training cohort and the test cohort were 0.922 and 0.875 (Figure 4a), respectively. Additionally, the AUC values for the whole sample (training cohort and test cohort) were higher than those for CA15‐3 and CA125, which are two commonly used tumor markers of breast cancer (AUC of 0.886 and 0.682, respectively) (Figure 4b).

Furthermore, in addition to its strong performance in distinguishing between breast cancer and non‐breast cancer cases, we also assessed its ability to differentiate between different subgroups in the test cohort. The AUC for breast cancer and HC was 0.920 (Figure 4c), while the AUC for breast cancer and benign cases was 0.91 (Figure 4d). However, the AUC for benign cases and the control group was comparatively lower. The findings of this study suggest that the combination of PON3, IGLV3‐10, and IGHV3‐73 proteins in a model demonstrates a significant ability to differentiate between individuals with breast cancer and HC.

## 4 Discussion

Breast cancer is a prevalent malignancy among women globally, holding the highest incidence rate among female malignant tumors. It stands as the primary cause of cancer‐related mortality in over 100 countries, posing a significant threat to women's physical and mental well‐being [20]. Research indicates that patients diagnosed with early‐stage non‐metastatic breast cancer exhibit a cure rate ranging from 70% to 80%. Conversely, patients with advanced breast cancer accompanied by distant organ metastasis lack recognized effective treatment options. Consequently, early detection, diagnosis, and treatment play a pivotal role in mitigating breast cancer mortality and improving prognosis. Despite the prevalent utilization of mammography as a screening modality for breast cancer, concerns persist regarding its propensity for generating false positive results, exposure to radiation, and the potential for overdiagnosis [21]. Consequently, there exists a pressing imperative to cultivate readily attainable, steadfast, and dependable biomarkers to facilitate non‐invasive screening and diagnosis of breast cancer.

CA15‐3 and CA125 are tumor biomarkers that possess considerable utility in breast cancer biomarker research. However, their sensitivity is limited and their specificity for local lesions is inadequate. Given the significant heterogeneity of breast cancer in terms of histology, epidemiology, and molecular characteristics, coupled with the diverse and intricate nature of tumor biomarkers, there is currently a lack of biomarkers suitable for early screening and diagnosis of breast cancer. In recent years, MS‐based high‐throughput and high‐precision proteomics technology has emerged as a valuable tool for elucidating the underlying mechanisms of breast cancer initiation and progression, identifying potential tumor markers and molecular targets associated with breast cancer, and facilitating early diagnosis, treatment, and prognosis assessment. Consequently, proteomics has progressively gained recognition as an effective approach in the realm of breast cancer research and clinical practice.

Luminal A breast cancer is the most common type of breast cancer. In our study, we selected plasma from Luminal A breast cancer patients, benign breast tumor patients, and HCs for proteomic analysis, and we analyzed and quantified 517 proteins, and significant protein expression differences were observed between breast cancer and HC, between benign and HC, and between breast cancer and benign, indicating that there was a specific protein expression profile in each case. Through functional annotation and enrichment analysis of differential proteins, we found that there were significant differences in lipid metabolism pathways in breast cancer and non‐breast cancer, suggesting that this pathway and the proteins involved in this pathway may play an important role in the pathophysiology of breast cancer.

We used the XGBoost machine learning model and AUC value to screen out the optimal combinations of variables for breast cancer diagnosis from a variety of differential proteins: PON3, IGLV3‐10, and IGHV3‐73. PON3 belongs to the paraoxonase family and is associated with high‐density lipoprotein (HDL) granules and exerts prominent anti‐inflammatory and antioxidant properties, mainly at the cellular level [22]. PON3 has also been shown to have hepatoprotective effects, preventing histological alterations and hepatocyte apoptosis that leads to liver disease [23]. PON3 is overexpressed in a variety of human cancers and has been shown to eliminate mitochondrial superoxide production, resulting in antioxidant and anti‐apoptotic benefits, saving tumor cells from death [24]. A meta‐analysis showed that PON3 was down‐regulated in hepatocellular carcinoma, renal clear cell sarcoma, ovarian serous papillary carcinoma, cervical cancer, papillary thyroid carcinoma, prostate cancer, and non‐Hodgkin lymphoma and up‐regulated in lung adenocarcinoma and pancreatic cancer [25, 26]. Studies have shown that PON3 can inhibit the migration and invasion of esophageal cancer cells [27], and inhibition of PON3 can significantly reduce the in vitro proliferation and metastasis of oral squamous cell carcinoma cells and slow down the progression of oral squamous cell carcinoma [28]. In this study, it was found that the expression of PON3 protein in the plasma of breast cancer patients was reduced, which was consistent with the previous results of breast cancer proteomics [29, 30]. It may be due to the fact that the decrease in PON3 activity negatively affects the antioxidant effect of the enzyme, thereby exposing the body to a higher state of oxidative stress, leading to cell damage, which may lead to carcinogenesis in the long term.

IGHV3‐10 and IGHV3‐73 are a subfamily of immunoglobulin heavy chain genes (IGHVs) involved in antigen recognition in the V region of the variable domain of immunoglobulin heavy chains, which are commonly used in the diagnosis of chronic lymphocytic leukemia [31]. We found that plasma expression levels of these two immune‐modulatory‐related proteins were also significantly reduced in breast cancer patients, which we hypothesized may be related to immune escape from tumors. These two proteins involved in adaptive immune responses have the potential to serve as markers for early diagnosis of breast cancer.

Our study identified a significant association between luminal A breast cancer and catabolic processes as well as cholesterol metabolism, corroborating the findings of previous research [32, 33]. Other research groups have investigated the differential expression of plasma proteins in patients with luminal A breast cancer compared to healthy individuals. In a study on breast cancer plasma proteomics conducted by Shankar Suman et al., it was found that in the luminal A subtype, FN protein exhibited an AUC of 0.745, with a sensitivity of 45% and a specificity of 95%. Similarly, CFB protein demonstrated an AUC of 0.750, with a sensitivity of 40% and a specificity of 100%, indicating the highest diagnostic efficiency [34]. The observed discrepancies in plasma markers between luminal A breast cancer patients in the present study and those reported in previous research may be attributed to variations in sample sources, research methodologies, and data analysis techniques. Although the sample size in the study conducted by Shankar Suman et al. (*N* = 8) was comparable to that of the present study (*N* = 10), our study employed rigorous screening criteria to include only luminal A breast cancer patients, benign breast tumor patients, and HCs. To identify biomarkers specific to luminal A breast cancer, we analyzed the differences between luminal A breast cancer patients and non‐breast cancer individuals (comprising patients with benign breast tumors and HCs) in the subsequent biomarker screening. Their study employed isobaric tag for absolute and relative quantitation (iTRAQ) quantitative proteomics, whereas our study utilized DIA proteomics. In comparison to iTRAQ, DIA proteomics offers the advantage of preserving low‐abundance protein information and demonstrates superior quantitative accuracy and data reproducibility. However, due to interindividual variability, inconsistent protein expression patterns are observed across different study populations, an issue that remains unavoidable.

Our study also has certain limitations, the first being its single‐center design and limited sample size. All of our participants were from a single center, and the sample size in each group was small due to strict screening criteria, and the results needed to be further validated in a large sample. The second limitation is the absence of tissue from breast cancer patients, which prevents us from assessing the differential expression of biomarkers in breast cancer tissue.

## 5 Conclusions

In conclusion, this study presents an analysis of systemic alterations in plasma protein profiles among patients with luminal A breast cancer. Additionally, we have successfully identified several plasma proteins that exhibit potential as biomarkers for luminal A breast cancer. It is important to note that these biomarkers are not presently accessible for clinical breast cancer screening. Nonetheless, our research serves as a valuable resource for further investigation into potential plasma protein diagnostic biomarkers for breast cancer. However, the limited sample size resulting from rigorous filtration necessitates a meticulous validation of the candidate proteins in subsequent investigations. We are confident that our findings will make a valuable contribution to the early detection of breast cancer and hold certain potential for clinical application in breast cancer screening.

## Author Contributions

**Meimei Zhao:** conceptualization (lead), formal analysis (lead), investigation (lead), methodology (lead), writing – review and editing (lead). **YongWei Jiang:** formal analysis (equal). **Xiaomu Kong:** methodology (equal). **Yi Liu:** methodology (equal). **Peng Gao:** formal analysis (equal). **Mo Li:** investigation (equal). **Haoyan Zhu:** investigation (equal). **Guoxiong Deng:** data curation (equal). **Ziyi Feng:** data curation (equal). **Yongtong Cao:** conceptualization (equal), supervision (equal), writing – review and editing (equal). **Liang Ma:** conceptualization (equal), supervision (equal).

## Ethics Statement

All procedures performed in studies involving human participants were executed in compliance with the Declaration of Helsinki II and received approval from the Ethics Committee of the China‐Japan Friendship Hospital.

## Consent

Informed consent was obtained from all individual participants included in the study. All authors have read and agreed to the published version of the manuscript.

## Conflicts of Interest

The authors declare no conflicts of interest.

## Supporting information


Data S1.

## Data Availability

All data generated or analyzed during this study are available from the first author (Meimei Zhao, zhaomeimei@bjmu.edu.cn) by request.
