# Supplementary material for: The Analysis of Plasma Proteomics for Luminal A Breast Cancer
Source: Cancer Med. 2024 Dec 6;13(23):e70470. doi: 10.1002/cam4.70470 (PMC11622152; doi:10.1002/cam4.70470)
Supplement: Supplementary file 1 — Data S1. [file CAM4-13-e70470-s001.docx]

**The analysis of plasma proteomics for Luminal A breast cancer**

Meimei Zhao^1^; YongWei Jiang^1^; Xiaomu Kong^1^; Yi Liu^1^; Peng Gao^1^; Mo Li^1^; Haoyan Zhu^1^; Guoxiong Deng^1^; Ziyi Feng^1^; Yongtong Cao^1^; Liang Ma^1^

1 Department of Clinical Laboratory, China-Japan Friendship Hospital, Beijing, China

*For Correspondence:

Liang Ma, Ph.D.; Yongtong Cao, Ph.D.,

Department of Clinical Laboratory, China-Japan Friendship Hospital, Beijing, China

Tel.: 008610-84206146

E-mail: caoyongtong100@sina.com; liangma321@aliyun.com

***Supplement Fig 1: Quality control of mass spectrometry.*** **a** Protein mass distribution map. **b** Distribution map of missed cutting sites. **c** Distribution map of peptide length. **d** Distribution map of peptide number. **e** Overview of the total sample protein. **f** Overview of the total sample peptides.


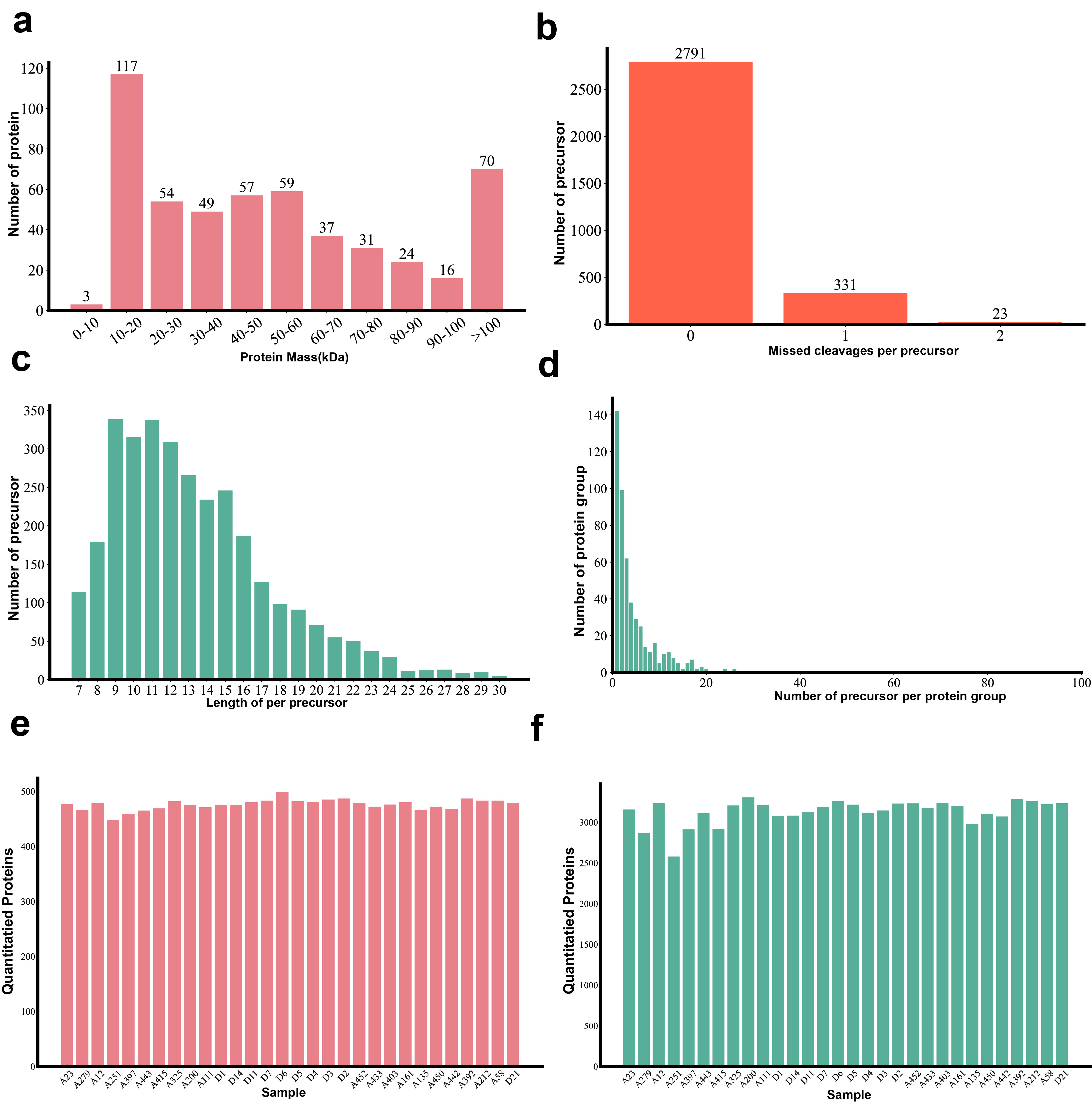


***Supplement Fig 2: Heat map and the volcano plot for quantitative difference of DEPs among each sample from*** ***breast cancer and*** ***benign.* a** Volcano map of differential expression of protein between breast cancer and benign. **b** Quantitative heat map of differential expression of protein in breast cancer and benign. **c** Principal component analysis (PCA) plot of BC group (green) and the benign group (red), each dot represents a sample.


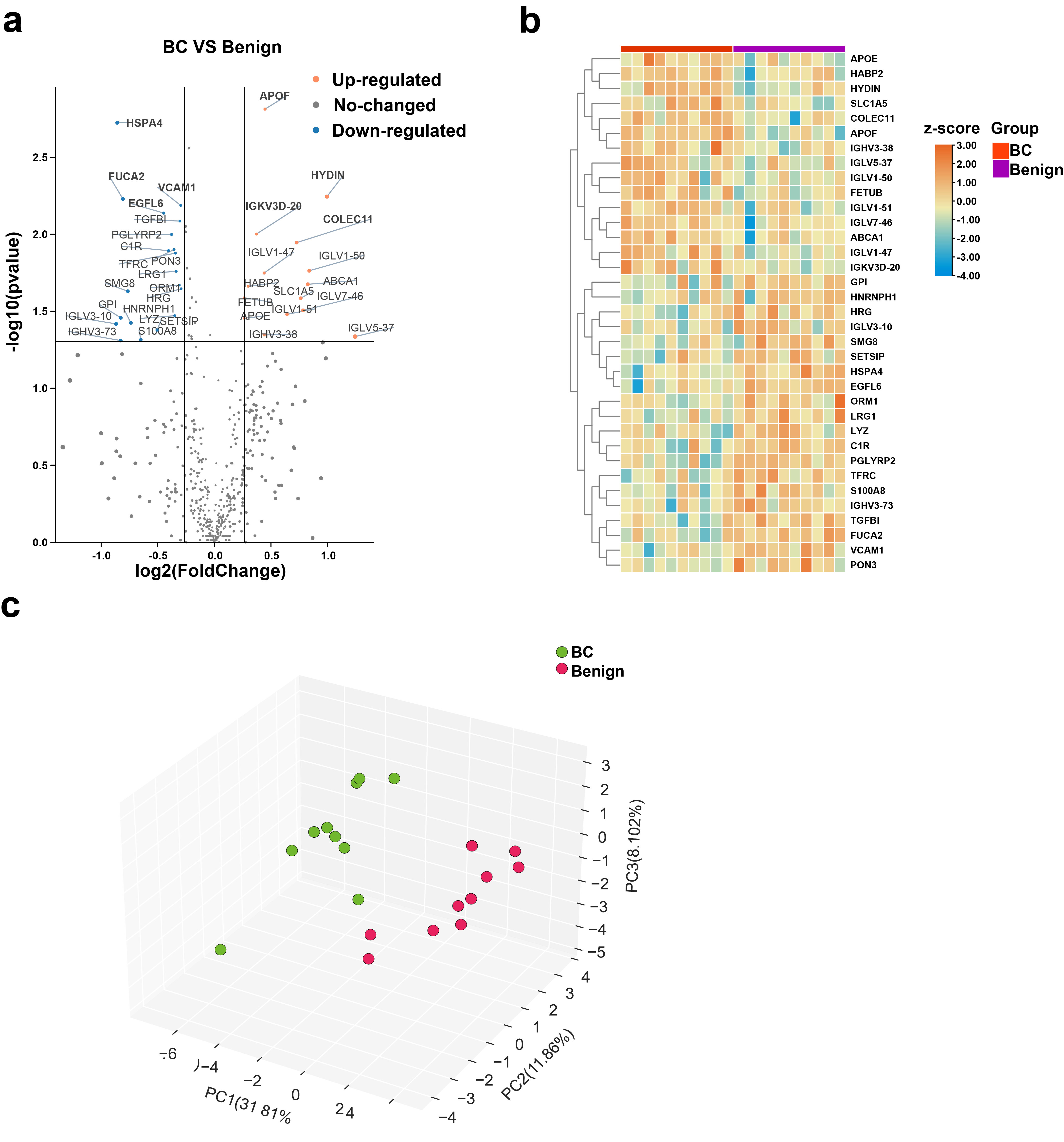


# *Table S1 3584 human proteins quantified in 30 samples*

| **Proteins** | **Protein Names** | **Genes** | **Description** | **Mol(kDa)** | **Length** |
| --- | --- | --- | --- | --- | --- |
| A0A075B6H7 | KV37_HUMAN | IGKV3-7 | Probable non-functional immunoglobulin kappa variable 3-7 OS=Homo sapiens OX=9606 GN=IGKV3-7 PE=1 SV=1 | 12.7835 | 116 |
| A0A075B6I0 | LV861_HUMAN | IGLV8-61 | Immunoglobulin lambda variable 8-61 OS=Homo sapiens OX=9606 GN=IGLV8-61 PE=3 SV=7 | 12.8144 | 122 |
| A0A075B6I6 | LV150_HUMAN | IGLV1-50 | Probable non-functional immunoglobulin lambda variable 1-50 OS=Homo sapiens OX=9606 GN=IGLV1-50 PE=1 SV=1 | 12.3387 | 118 |
| A0A075B6I9 | LV746_HUMAN | IGLV7-46 | Immunoglobulin lambda variable 7-46 OS=Homo sapiens OX=9606 GN=IGLV7-46 PE=3 SV=4 | 12.4681 | 117 |
| A0A075B6J1 | LV537_HUMAN | IGLV5-37 | Immunoglobulin lambda variable 5-37 OS=Homo sapiens OX=9606 GN=IGLV5-37 PE=3 SV=1 | 13.2768 | 123 |
| A0A075B6J2 | LV233_HUMAN | IGLV2-33 | Probable non-functional immunoglobulin lambda variable 2-33 OS=Homo sapiens OX=9606 GN=IGLV2-33 PE=1 SV=2 | 12.6862 | 118 |
| A0A075B6J9 | LV218_HUMAN | IGLV2-18 | Immunoglobulin lambda variable 2-18 OS=Homo sapiens OX=9606 GN=IGLV2-18 PE=3 SV=2 | 12.4117 | 118 |
| A0A075B6K4 | LV310_HUMAN | IGLV3-10 | Immunoglobulin lambda variable 3-10 OS=Homo sapiens OX=9606 GN=IGLV3-10 PE=3 SV=2 | 12.4409 | 115 |
| A0A075B6K5 | LV39_HUMAN | IGLV3-9 | Immunoglobulin lambda variable 3-9 OS=Homo sapiens OX=9606 GN=IGLV3-9 PE=3 SV=1 | 12.3317 | 115 |
| A0A075B6P5 | KV228_HUMAN | IGKV2-28 | Immunoglobulin kappa variable 2-28 OS=Homo sapiens OX=9606 GN=IGKV2-28 PE=3 SV=1 | 12.9568 | 120 |
| A0A075B6Q5 | HV364_HUMAN | IGHV3-64 | Immunoglobulin heavy variable 3-64 OS=Homo sapiens OX=9606 GN=IGHV3-64 PE=3 SV=1 | 12.8907 | 118 |
| A0A075B6R2 | HV404_HUMAN | IGHV4-4 | Immunoglobulin heavy variable 4-4 OS=Homo sapiens OX=9606 GN=IGHV4-4 PE=3 SV=2 | 12.8477 | 117 |
| A0A075B6R9 | KVD24_HUMAN | IGKV2D-24 | Probable non-functional immunoglobulin kappa variable 2D-24 OS=Homo sapiens OX=9606 GN=IGKV2D-24 PE=1 SV=1 | 13.0789 | 120 |
| A0A075B6S2 | KVD29_HUMAN | IGKV2D-29 | Immunoglobulin kappa variable 2D-29 OS=Homo sapiens OX=9606 GN=IGKV2D-29 PE=3 SV=1 | 13.1431 | 120 |
| A0A075B6S5 | KV127_HUMAN | IGKV1-27 | Immunoglobulin kappa variable 1-27 OS=Homo sapiens OX=9606 GN=IGKV1-27 PE=3 SV=1 | 12.7115 | 117 |
| A0A075B6S6 | KVD30_HUMAN | IGKV2D-30 | Immunoglobulin kappa variable 2D-30 OS=Homo sapiens OX=9606 GN=IGKV2D-30 PE=3 SV=1 | 13.215 | 120 |
| A0A087WSX0 | LV545_HUMAN | IGLV5-45 | Immunoglobulin lambda variable 5-45 OS=Homo sapiens OX=9606 GN=IGLV5-45 PE=3 SV=1 | 13.1618 | 123 |
| A0A087WSY6 | KVD15_HUMAN | IGKV3D-15 | Immunoglobulin kappa variable 3D-15 OS=Homo sapiens OX=9606 GN=IGKV3D-15 PE=3 SV=6 | 12.5342 | 115 |
| A0A087WSZ0 | KVD08_HUMAN | IGKV1D-8 | Immunoglobulin kappa variable 1D-8 OS=Homo sapiens OX=9606 GN=IGKV1D-8 PE=3 SV=6 | 12.8367 | 117 |
| A0A087WW87 | KV240_HUMAN | IGKV2-40 | Immunoglobulin kappa variable 2-40 OS=Homo sapiens OX=9606 GN=IGKV2-40 PE=3 SV=2 | 13.3101 | 121 |
| A0A0A0MRZ8 | KVD11_HUMAN | IGKV3D-11 | Immunoglobulin kappa variable 3D-11 OS=Homo sapiens OX=9606 GN=IGKV3D-11 PE=3 SV=6 | 12.6252 | 115 |
| A0A0A0MS00 | LV332_HUMAN | IGLV3-32 | Probable non-functional immunoglobulin lambda variable 3-32 OS=Homo sapiens OX=9606 GN=IGLV3-32 PE=1 SV=5 | 12.3548 | 114 |
| A0A0A0MS14 | HV145_HUMAN | IGHV1-45 | Immunoglobulin heavy variable 1-45 OS=Homo sapiens OX=9606 GN=IGHV1-45 PE=3 SV=1 | 13.5084 | 117 |
| A0A0A0MS15 | HV349_HUMAN | IGHV3-49 | Immunoglobulin heavy variable 3-49 OS=Homo sapiens OX=9606 GN=IGHV3-49 PE=3 SV=1 | 13.0559 | 119 |
| A0A0A0MT36 | KVD21_HUMAN | IGKV6D-21 | Immunoglobulin kappa variable 6D-21 OS=Homo sapiens OX=9606 GN=IGKV6D-21 PE=3 SV=1 | 12.3399 | 114 |
| A0A0B4J1U3 | LV136_HUMAN | IGLV1-36 | Immunoglobulin lambda variable 1-36 OS=Homo sapiens OX=9606 GN=IGLV1-36 PE=1 SV=5 | 12.4778 | 117 |
| A0A0B4J1U7 | HV601_HUMAN | IGHV6-1 | Immunoglobulin heavy variable 6-1 OS=Homo sapiens OX=9606 GN=IGHV6-1 PE=3 SV=1 | 13.4813 | 121 |
| A0A0B4J1V0 | HV315_HUMAN | IGHV3-15 | Immunoglobulin heavy variable 3-15 OS=Homo sapiens OX=9606 GN=IGHV3-15 PE=3 SV=1 | 12.9258 | 119 |
| A0A0B4J1V2 | HV226_HUMAN | IGHV2-26 | Immunoglobulin heavy variable 2-26 OS=Homo sapiens OX=9606 GN=IGHV2-26 PE=3 SV=1 | 13.1823 | 119 |
| A0A0B4J1V6 | HV373_HUMAN | IGHV3-73 | Immunoglobulin heavy variable 3-73 OS=Homo sapiens OX=9606 GN=IGHV3-73 PE=3 SV=1 | 12.8577 | 119 |
| A0A0B4J1X5 | HV374_HUMAN | IGHV3-74 | Immunoglobulin heavy variable 3-74 OS=Homo sapiens OX=9606 GN=IGHV3-74 PE=3 SV=1 | 12.8396 | 117 |
| A0A0B4J1X8 | HV343_HUMAN | IGHV3-43 | Immunoglobulin heavy variable 3-43 OS=Homo sapiens OX=9606 GN=IGHV3-43 PE=3 SV=1 | 13.0768 | 118 |
| A0A0B4J1Y8 | LV949_HUMAN | IGLV9-49 | Immunoglobulin lambda variable 9-49 OS=Homo sapiens OX=9606 GN=IGLV9-49 PE=1 SV=1 | 13.0237 | 123 |
| A0A0B4J1Y9 | HV372_HUMAN | IGHV3-72 | Immunoglobulin heavy variable 3-72 OS=Homo sapiens OX=9606 GN=IGHV3-72 PE=3 SV=1 | 13.2029 | 119 |
| A0A0B4J2D9 | KVD13_HUMAN | IGKV1D-13 | Immunoglobulin kappa variable 1D-13 OS=Homo sapiens OX=9606 GN=IGKV1D-13 PE=3 SV=1 | 12.5693 | 117 |
| A0A0B4J2H0 | HV69D_HUMAN | IGHV1-69D | Immunoglobulin heavy variable 1-69D OS=Homo sapiens OX=9606 GN=IGHV1-69D PE=1 SV=1 | 12.6603 | 117 |
| A0A0C4DH24 | KV621_HUMAN | IGKV6-21 | Immunoglobulin kappa variable 6-21 OS=Homo sapiens OX=9606 GN=IGKV6-21 PE=3 SV=1 | 12.43 | 114 |
| A0A0C4DH25 | KVD20_HUMAN | IGKV3D-20 | Immunoglobulin kappa variable 3D-20 OS=Homo sapiens OX=9606 GN=IGKV3D-20 PE=3 SV=1 | 12.5151 | 116 |
| A0A0C4DH29 | HV103_HUMAN | IGHV1-3 | Immunoglobulin heavy variable 1-3 OS=Homo sapiens OX=9606 GN=IGHV1-3 PE=3 SV=1 | 13.0077 | 117 |
| A0A0C4DH31 | HV118_HUMAN | IGHV1-18 | Immunoglobulin heavy variable 1-18 OS=Homo sapiens OX=9606 GN=IGHV1-18 PE=3 SV=1 | 12.8204 | 117 |
| A0A0C4DH33 | HV124_HUMAN | IGHV1-24 | Immunoglobulin heavy variable 1-24 OS=Homo sapiens OX=9606 GN=IGHV1-24 PE=3 SV=1 | 12.8245 | 117 |
| A0A0C4DH35 | HV335_HUMAN | IGHV3-35 | Probable non-functional immunoglobulin heavy variable 3-35 OS=Homo sapiens OX=9606 GN=IGHV3-35 PE=1 SV=1 | 12.8104 | 117 |
| A0A0C4DH36 | HV338_HUMAN | IGHV3-38 | Probable non-functional immunoglobulin heavy variable 3-38 OS=Homo sapiens OX=9606 GN=IGHV3-38 PE=1 SV=1 | 12.7585 | 116 |
| A0A0C4DH38 | HV551_HUMAN | IGHV5-51 | Immunoglobulin heavy variable 5-51 OS=Homo sapiens OX=9606 GN=IGHV5-51 PE=3 SV=1 | 12.6746 | 117 |
| A0A0C4DH67 | KV108_HUMAN | IGKV1-8 | Immunoglobulin kappa variable 1-8 OS=Homo sapiens OX=9606 GN=IGKV1-8 PE=3 SV=1 | 12.5373 | 115 |
| A0A0C4DH73 | KV112_HUMAN | IGKV1-12 | Immunoglobulin kappa variable 1-12 OS=Homo sapiens OX=9606 GN=IGKV1-12 PE=3 SV=1 | 12.6454 | 117 |
| A0A0J9YX35 | HV64D_HUMAN | IGHV3-64D | Immunoglobulin heavy variable 3-64D OS=Homo sapiens OX=9606 GN=IGHV3-64D PE=3 SV=1 | 12.8226 | 117 |
| A0A0J9YXX1 | HV5X1_HUMAN | IGHV5-10-1 | Immunoglobulin heavy variable 5-10-1 OS=Homo sapiens OX=9606 GN=IGHV5-10-1 PE=3 SV=1 | 12.7726 | 117 |
| A0A2R8Y619 | H2BE1_HUMAN | H2BE1 | Histone H2B type 2-E1 OS=Homo sapiens OX=9606 GN=H2BE1 PE=3 SV=1 | 13.4915 | 122 |
| A2NJV5 | KV229_HUMAN | IGKV2-29 | Immunoglobulin kappa variable 2-29 OS=Homo sapiens OX=9606 GN=IGKV2-29 PE=3 SV=2 | 13.0851 | 120 |
| B2RXH8 | HNRC2_HUMAN | HNRNPCL2 | Heterogeneous nuclear ribonucleoprotein C-like 2 OS=Homo sapiens OX=9606 GN=HNRNPCL2 PE=1 SV=1 | 32.0723 | 293 |
| O00151 | PDLI1_HUMAN | PDLIM1 | PDZ and LIM domain protein 1 OS=Homo sapiens OX=9606 GN=PDLIM1 PE=1 SV=4 | 36.0717 | 329 |
| O00187 | MASP2_HUMAN | MASP2 | Mannan-binding lectin serine protease 2 OS=Homo sapiens OX=9606 GN=MASP2 PE=1 SV=4 | 75.7024 | 686 |
| O00391 | QSOX1_HUMAN | QSOX1 | Sulfhydryl oxidase 1 OS=Homo sapiens OX=9606 GN=QSOX1 PE=1 SV=3 | 82.5777 | 747 |
| O00533 | NCHL1_HUMAN | CHL1 | Neural cell adhesion molecule L1-like protein OS=Homo sapiens OX=9606 GN=CHL1 PE=1 SV=4 | 135.0715 | 1208 |
| O00571 | DDX3X_HUMAN | DDX3X | ATP-dependent RNA helicase DDX3X OS=Homo sapiens OX=9606 GN=DDX3X PE=1 SV=3 | 73.2434 | 662 |
| O14786 | NRP1_HUMAN | NRP1 | Neuropilin-1 OS=Homo sapiens OX=9606 GN=NRP1 PE=1 SV=3 | 103.1343 | 923 |
| O14791 | APOL1_HUMAN | APOL1 | Apolipoprotein L1 OS=Homo sapiens OX=9606 GN=APOL1 PE=1 SV=5 | 43.9742 | 398 |
| O15021 | MAST4_HUMAN | MAST4 | Microtubule-associated serine/threonine-protein kinase 4 OS=Homo sapiens OX=9606 GN=MAST4 PE=1 SV=4 | 284.0973 | 2623 |
| O15212 | PFD6_HUMAN | PFDN6 | Prefoldin subunit 6 OS=Homo sapiens OX=9606 GN=PFDN6 PE=1 SV=1 | 14.5827 | 129 |
| O43390 | HNRPR_HUMAN | HNRNPR | Heterogeneous nuclear ribonucleoprotein R OS=Homo sapiens OX=9606 GN=HNRNPR PE=1 SV=1 | 70.9431 | 633 |
| O43707 | ACTN4_HUMAN | ACTN4 | Alpha-actinin-4 OS=Homo sapiens OX=9606 GN=ACTN4 PE=1 SV=2 | 104.854 | 911 |
| O43866 | CD5L_HUMAN | CD5L | CD5 antigen-like OS=Homo sapiens OX=9606 GN=CD5L PE=1 SV=1 | 38.0878 | 347 |
| O60506 | HNRPQ_HUMAN | SYNCRIP | Heterogeneous nuclear ribonucleoprotein Q OS=Homo sapiens OX=9606 GN=SYNCRIP PE=1 SV=2 | 69.6026 | 623 |
| O75015 | FCG3B_HUMAN | FCGR3B | Low affinity immunoglobulin gamma Fc region receptor III-B OS=Homo sapiens OX=9606 GN=FCGR3B PE=1 SV=2 | 26.2159 | 233 |
| O75144 | ICOSL_HUMAN | ICOSLG | ICOS ligand OS=Homo sapiens OX=9606 GN=ICOSLG PE=1 SV=2 | 33.3488 | 302 |
| O75636 | FCN3_HUMAN | FCN3 | Ficolin-3 OS=Homo sapiens OX=9606 GN=FCN3 PE=1 SV=2 | 32.903 | 299 |
| O75882 | ATRN_HUMAN | ATRN | Attractin OS=Homo sapiens OX=9606 GN=ATRN PE=1 SV=2 | 158.5369 | 1429 |
| O95445 | APOM_HUMAN | APOM | Apolipoprotein M OS=Homo sapiens OX=9606 GN=APOM PE=1 SV=2 | 21.2533 | 188 |
| O95477 | ABCA1_HUMAN | ABCA1 | Phospholipid-transporting ATPase ABCA1 OS=Homo sapiens OX=9606 GN=ABCA1 PE=1 SV=3 | 254.3021 | 2261 |
| O95497 | VNN1_HUMAN | VNN1 | Pantetheinase OS=Homo sapiens OX=9606 GN=VNN1 PE=1 SV=2 | 57.0117 | 513 |
| O95980 | RECK_HUMAN | RECK | Reversion-inducing cysteine-rich protein with Kazal motifs OS=Homo sapiens OX=9606 GN=RECK PE=1 SV=1 | 106.4573 | 971 |
| P00338 | LDHA_HUMAN | LDHA | L-lactate dehydrogenase A chain OS=Homo sapiens OX=9606 GN=LDHA PE=1 SV=2 | 36.6887 | 332 |
| P00450 | CERU_HUMAN | CP | Ceruloplasmin OS=Homo sapiens OX=9606 GN=CP PE=1 SV=1 | 122.2052 | 1065 |
| P00488 | F13A_HUMAN | F13A1 | Coagulation factor XIII A chain OS=Homo sapiens OX=9606 GN=F13A1 PE=1 SV=4 | 83.2673 | 732 |
| P00558 | PGK1_HUMAN | PGK1 | Phosphoglycerate kinase 1 OS=Homo sapiens OX=9606 GN=PGK1 PE=1 SV=3 | 44.6147 | 417 |
| P00734 | THRB_HUMAN | F2 | Prothrombin OS=Homo sapiens OX=9606 GN=F2 PE=1 SV=2 | 70.0369 | 622 |
| P00736 | C1R_HUMAN | C1R | Complement C1r subcomponent OS=Homo sapiens OX=9606 GN=C1R PE=1 SV=2 | 80.1186 | 705 |
| P00738 | HPT_HUMAN | HP | Haptoglobin OS=Homo sapiens OX=9606 GN=HP PE=1 SV=1 | 45.2053 | 406 |
| P00739 | HPTR_HUMAN | HPR | Haptoglobin-related protein OS=Homo sapiens OX=9606 GN=HPR PE=2 SV=2 | 39.0296 | 348 |
| P00740 | FA9_HUMAN | F9 | Coagulation factor IX OS=Homo sapiens OX=9606 GN=F9 PE=1 SV=2 | 51.7784 | 461 |
| P00742 | FA10_HUMAN | F10 | Coagulation factor X OS=Homo sapiens OX=9606 GN=F10 PE=1 SV=2 | 54.7317 | 488 |
| P00746 | CFAD_HUMAN | CFD | Complement factor D OS=Homo sapiens OX=9606 GN=CFD PE=1 SV=5 | 27.0329 | 253 |
| P00747 | PLMN_HUMAN | PLG | Plasminogen OS=Homo sapiens OX=9606 GN=PLG PE=1 SV=2 | 90.569 | 810 |
| P00748 | FA12_HUMAN | F12 | Coagulation factor XII OS=Homo sapiens OX=9606 GN=F12 PE=1 SV=3 | 67.7921 | 615 |
| P00749 | UROK_HUMAN | PLAU | Urokinase-type plasminogen activator OS=Homo sapiens OX=9606 GN=PLAU PE=1 SV=2 | 48.5074 | 431 |
| P00751 | CFAB_HUMAN | CFB | Complement factor B OS=Homo sapiens OX=9606 GN=CFB PE=1 SV=2 | 85.5329 | 764 |
| P00915 | CAH1_HUMAN | CA1 | Carbonic anhydrase 1 OS=Homo sapiens OX=9606 GN=CA1 PE=1 SV=2 | 28.8702 | 261 |
| P00918 | CAH2_HUMAN | CA2 | Carbonic anhydrase 2 OS=Homo sapiens OX=9606 GN=CA2 PE=1 SV=2 | 29.2461 | 260 |
| P00966 | ASSY_HUMAN | ASS1 | Argininosuccinate synthase OS=Homo sapiens OX=9606 GN=ASS1 PE=1 SV=2 | 46.5304 | 412 |
| P01008 | ANT3_HUMAN | SERPINC1 | Antithrombin-III OS=Homo sapiens OX=9606 GN=SERPINC1 PE=1 SV=1 | 52.6024 | 464 |
| P01009 | A1AT_HUMAN | SERPINA1 | Alpha-1-antitrypsin OS=Homo sapiens OX=9606 GN=SERPINA1 PE=1 SV=3 | 46.7366 | 418 |
| P01011 | AACT_HUMAN | SERPINA3 | Alpha-1-antichymotrypsin OS=Homo sapiens OX=9606 GN=SERPINA3 PE=1 SV=2 | 47.6509 | 423 |
| P01019 | ANGT_HUMAN | AGT | Angiotensinogen OS=Homo sapiens OX=9606 GN=AGT PE=1 SV=1 | 53.1542 | 485 |
| P01023 | A2MG_HUMAN | A2M | Alpha-2-macroglobulin OS=Homo sapiens OX=9606 GN=A2M PE=1 SV=3 | 163.291 | 1474 |
| P01024 | CO3_HUMAN | C3 | Complement C3 OS=Homo sapiens OX=9606 GN=C3 PE=1 SV=2 | 187.1481 | 1663 |
| P01031 | CO5_HUMAN | C5 | Complement C5 OS=Homo sapiens OX=9606 GN=C5 PE=1 SV=4 | 188.3053 | 1676 |
| P01033 | TIMP1_HUMAN | TIMP1 | Metalloproteinase inhibitor 1 OS=Homo sapiens OX=9606 GN=TIMP1 PE=1 SV=1 | 23.1709 | 207 |
| P01034 | CYTC_HUMAN | CST3 | Cystatin-C OS=Homo sapiens OX=9606 GN=CST3 PE=1 SV=1 | 15.7992 | 146 |
| P01042 | KNG1_HUMAN | KNG1 | Kininogen-1 OS=Homo sapiens OX=9606 GN=KNG1 PE=1 SV=2 | 71.9574 | 644 |
| P01137 | TGFB1_HUMAN | TGFB1 | Transforming growth factor beta-1 proprotein OS=Homo sapiens OX=9606 GN=TGFB1 PE=1 SV=2 | 44.3412 | 390 |
| P01591 | IGJ_HUMAN | JCHAIN | Immunoglobulin J chain OS=Homo sapiens OX=9606 GN=JCHAIN PE=1 SV=4 | 18.0986 | 159 |
| P01593 | KVD33_HUMAN | IGKV1D-33 | Immunoglobulin kappa variable 1D-33 OS=Homo sapiens OX=9606 GN=IGKV1D-33 PE=1 SV=2 | 12.8485 | 117 |
| P01597 | KV139_HUMAN | IGKV1-39 | Immunoglobulin kappa variable 1-39 OS=Homo sapiens OX=9606 GN=IGKV1-39 PE=1 SV=2 | 12.7375 | 117 |
| P01599 | KV117_HUMAN | IGKV1-17 | Immunoglobulin kappa variable 1-17 OS=Homo sapiens OX=9606 GN=IGKV1-17 PE=1 SV=2 | 12.7785 | 117 |
| P01602 | KV105_HUMAN | IGKV1-5 | Immunoglobulin kappa variable 1-5 OS=Homo sapiens OX=9606 GN=IGKV1-5 PE=1 SV=2 | 12.7816 | 117 |
| P01619 | KV320_HUMAN | IGKV3-20 | Immunoglobulin kappa variable 3-20 OS=Homo sapiens OX=9606 GN=IGKV3-20 PE=1 SV=2 | 12.5571 | 116 |
| P01700 | LV147_HUMAN | IGLV1-47 | Immunoglobulin lambda variable 1-47 OS=Homo sapiens OX=9606 GN=IGLV1-47 PE=1 SV=2 | 12.2836 | 117 |
| P01701 | LV151_HUMAN | IGLV1-51 | Immunoglobulin lambda variable 1-51 OS=Homo sapiens OX=9606 GN=IGLV1-51 PE=1 SV=2 | 12.2487 | 117 |
| P01706 | LV211_HUMAN | IGLV2-11 | Immunoglobulin lambda variable 2-11 OS=Homo sapiens OX=9606 GN=IGLV2-11 PE=1 SV=2 | 12.644 | 119 |
| P01709 | LV208_HUMAN | IGLV2-8 | Immunoglobulin lambda variable 2-8 OS=Homo sapiens OX=9606 GN=IGLV2-8 PE=1 SV=2 | 12.3816 | 118 |
| P01714 | LV319_HUMAN | IGLV3-19 | Immunoglobulin lambda variable 3-19 OS=Homo sapiens OX=9606 GN=IGLV3-19 PE=1 SV=2 | 12.0424 | 112 |
| P01717 | LV325_HUMAN | IGLV3-25 | Immunoglobulin lambda variable 3-25 OS=Homo sapiens OX=9606 GN=IGLV3-25 PE=1 SV=2 | 12.0114 | 112 |
| P01721 | LV657_HUMAN | IGLV6-57 | Immunoglobulin lambda variable 6-57 OS=Homo sapiens OX=9606 GN=IGLV6-57 PE=1 SV=2 | 12.5657 | 117 |
| P01742 | HV169_HUMAN | IGHV1-69 | Immunoglobulin heavy variable 1-69 OS=Homo sapiens OX=9606 GN=IGHV1-69 PE=1 SV=2 | 12.6594 | 117 |
| P01743 | HV146_HUMAN | IGHV1-46 | Immunoglobulin heavy variable 1-46 OS=Homo sapiens OX=9606 GN=IGHV1-46 PE=1 SV=2 | 12.9327 | 117 |
| P01764 | HV323_HUMAN | IGHV3-23 | Immunoglobulin heavy variable 3-23 OS=Homo sapiens OX=9606 GN=IGHV3-23 PE=1 SV=2 | 12.5823 | 117 |
| P01768 | HV330_HUMAN | IGHV3-30 | Immunoglobulin heavy variable 3-30 OS=Homo sapiens OX=9606 GN=IGHV3-30 PE=1 SV=2 | 12.9468 | 117 |
| P01780 | HV307_HUMAN | IGHV3-7 | Immunoglobulin heavy variable 3-7 OS=Homo sapiens OX=9606 GN=IGHV3-7 PE=1 SV=2 | 12.9427 | 117 |
| P01782 | HV309_HUMAN | IGHV3-9 | Immunoglobulin heavy variable 3-9 OS=Homo sapiens OX=9606 GN=IGHV3-9 PE=1 SV=2 | 12.9448 | 118 |
| P01833 | PIGR_HUMAN | PIGR | Polymeric immunoglobulin receptor OS=Homo sapiens OX=9606 GN=PIGR PE=1 SV=4 | 83.2836 | 764 |
| P01834 | IGKC_HUMAN | IGKC | Immunoglobulin kappa constant OS=Homo sapiens OX=9606 GN=IGKC PE=1 SV=2 | 11.765 | 107 |
| P01859 | IGHG2_HUMAN | IGHG2 | Immunoglobulin heavy constant gamma 2 OS=Homo sapiens OX=9606 GN=IGHG2 PE=1 SV=2 | 35.9006 | 326 |
| P01860 | IGHG3_HUMAN | IGHG3 | Immunoglobulin heavy constant gamma 3 OS=Homo sapiens OX=9606 GN=IGHG3 PE=1 SV=2 | 41.287 | 377 |
| P01861 | IGHG4_HUMAN | IGHG4 | Immunoglobulin heavy constant gamma 4 OS=Homo sapiens OX=9606 GN=IGHG4 PE=1 SV=1 | 35.9406 | 327 |
| P01871 | IGHM_HUMAN | IGHM | Immunoglobulin heavy constant mu OS=Homo sapiens OX=9606 GN=IGHM PE=1 SV=4 | 49.4397 | 453 |
| P01876 | IGHA1_HUMAN | IGHA1 | Immunoglobulin heavy constant alpha 1 OS=Homo sapiens OX=9606 GN=IGHA1 PE=1 SV=2 | 37.6547 | 353 |
| P02042 | HBD_HUMAN | HBD | Hemoglobin subunit delta OS=Homo sapiens OX=9606 GN=HBD PE=1 SV=2 | 16.0555 | 147 |
| P02533 | K1C14_HUMAN | KRT14 | Keratin, type I cytoskeletal 14 OS=Homo sapiens OX=9606 GN=KRT14 PE=1 SV=4 | 51.5615 | 472 |
| P02538 | K2C6A_HUMAN | KRT6A | Keratin, type II cytoskeletal 6A OS=Homo sapiens OX=9606 GN=KRT6A PE=1 SV=3 | 60.045 | 564 |
| P02647 | APOA1_HUMAN | APOA1 | Apolipoprotein A-I OS=Homo sapiens OX=9606 GN=APOA1 PE=1 SV=1 | 30.7778 | 267 |
| P02649 | APOE_HUMAN | APOE | Apolipoprotein E OS=Homo sapiens OX=9606 GN=APOE PE=1 SV=1 | 36.1541 | 317 |
| P02652 | APOA2_HUMAN | APOA2 | Apolipoprotein A-II OS=Homo sapiens OX=9606 GN=APOA2 PE=1 SV=1 | 11.175 | 100 |
| P02654 | APOC1_HUMAN | APOC1 | Apolipoprotein C-I OS=Homo sapiens OX=9606 GN=APOC1 PE=1 SV=1 | 9.3319 | 83 |
| P02655 | APOC2_HUMAN | APOC2 | Apolipoprotein C-II OS=Homo sapiens OX=9606 GN=APOC2 PE=1 SV=1 | 11.2839 | 101 |
| P02656 | APOC3_HUMAN | APOC3 | Apolipoprotein C-III OS=Homo sapiens OX=9606 GN=APOC3 PE=1 SV=1 | 10.8523 | 99 |
| P02671 | FIBA_HUMAN | FGA | Fibrinogen alpha chain OS=Homo sapiens OX=9606 GN=FGA PE=1 SV=2 | 94.973 | 866 |
| P02675 | FIBB_HUMAN | FGB | Fibrinogen beta chain OS=Homo sapiens OX=9606 GN=FGB PE=1 SV=2 | 55.9282 | 491 |
| P02679 | FIBG_HUMAN | FGG | Fibrinogen gamma chain OS=Homo sapiens OX=9606 GN=FGG PE=1 SV=3 | 51.5117 | 453 |
| P02730 | B3AT_HUMAN | SLC4A1 | Band 3 anion transport protein OS=Homo sapiens OX=9606 GN=SLC4A1 PE=1 SV=3 | 101.7923 | 911 |
| P02741 | CRP_HUMAN | CRP | C-reactive protein OS=Homo sapiens OX=9606 GN=CRP PE=1 SV=1 | 25.0386 | 224 |
| P02743 | SAMP_HUMAN | APCS | Serum amyloid P-component OS=Homo sapiens OX=9606 GN=APCS PE=1 SV=2 | 25.3871 | 223 |
| P02745 | C1QA_HUMAN | C1QA | Complement C1q subcomponent subunit A OS=Homo sapiens OX=9606 GN=C1QA PE=1 SV=2 | 26.0166 | 245 |
| P02746 | C1QB_HUMAN | C1QB | Complement C1q subcomponent subunit B OS=Homo sapiens OX=9606 GN=C1QB PE=1 SV=3 | 26.7218 | 253 |
| P02747 | C1QC_HUMAN | C1QC | Complement C1q subcomponent subunit C OS=Homo sapiens OX=9606 GN=C1QC PE=1 SV=3 | 25.7737 | 245 |
| P02748 | CO9_HUMAN | C9 | Complement component C9 OS=Homo sapiens OX=9606 GN=C9 PE=1 SV=2 | 63.1734 | 559 |
| P02749 | APOH_HUMAN | APOH | Beta-2-glycoprotein 1 OS=Homo sapiens OX=9606 GN=APOH PE=1 SV=3 | 38.2982 | 345 |
| P02750 | A2GL_HUMAN | LRG1 | Leucine-rich alpha-2-glycoprotein OS=Homo sapiens OX=9606 GN=LRG1 PE=1 SV=2 | 38.1779 | 347 |
| P02751 | FINC_HUMAN | FN1 | Fibronectin OS=Homo sapiens OX=9606 GN=FN1 PE=1 SV=5 | 272.3204 | 2477 |
| P02753 | RET4_HUMAN | RBP4 | Retinol-binding protein 4 OS=Homo sapiens OX=9606 GN=RBP4 PE=1 SV=3 | 23.01 | 201 |
| P02760 | AMBP_HUMAN | AMBP | Protein AMBP OS=Homo sapiens OX=9606 GN=AMBP PE=1 SV=1 | 38.9995 | 352 |
| P02763 | A1AG1_HUMAN | ORM1 | Alpha-1-acid glycoprotein 1 OS=Homo sapiens OX=9606 GN=ORM1 PE=1 SV=1 | 23.5116 | 201 |
| P02765 | FETUA_HUMAN | AHSG | Alpha-2-HS-glycoprotein OS=Homo sapiens OX=9606 GN=AHSG PE=1 SV=2 | 39.3407 | 367 |
| P02766 | TTHY_HUMAN | TTR | Transthyretin OS=Homo sapiens OX=9606 GN=TTR PE=1 SV=1 | 15.887 | 147 |
| P02768 | ALBU_HUMAN | ALB | Albumin OS=Homo sapiens OX=9606 GN=ALB PE=1 SV=2 | 69.3667 | 609 |
| P02774 | VTDB_HUMAN | GC | Vitamin D-binding protein OS=Homo sapiens OX=9606 GN=GC PE=1 SV=2 | 52.9175 | 474 |
| P02775 | CXCL7_HUMAN | PPBP | Platelet basic protein OS=Homo sapiens OX=9606 GN=PPBP PE=1 SV=3 | 13.8942 | 128 |
| P02776 | PLF4_HUMAN | PF4 | Platelet factor 4 OS=Homo sapiens OX=9606 GN=PF4 PE=1 SV=2 | 10.8449 | 101 |
| P02786 | TFR1_HUMAN | TFRC | Transferrin receptor protein 1 OS=Homo sapiens OX=9606 GN=TFRC PE=1 SV=2 | 84.8714 | 760 |
| P02787 | TRFE_HUMAN | TF | Serotransferrin OS=Homo sapiens OX=9606 GN=TF PE=1 SV=3 | 77.0639 | 698 |
| P02788 | TRFL_HUMAN | LTF | Lactotransferrin OS=Homo sapiens OX=9606 GN=LTF PE=1 SV=6 | 78.182 | 710 |
| P02790 | HEMO_HUMAN | HPX | Hemopexin OS=Homo sapiens OX=9606 GN=HPX PE=1 SV=2 | 51.6764 | 462 |
| P03950 | ANGI_HUMAN | ANG | Angiogenin OS=Homo sapiens OX=9606 GN=ANG PE=1 SV=1 | 16.5501 | 147 |
| P03951 | FA11_HUMAN | F11 | Coagulation factor XI OS=Homo sapiens OX=9606 GN=F11 PE=1 SV=1 | 70.1091 | 625 |
| P03952 | KLKB1_HUMAN | KLKB1 | Plasma kallikrein OS=Homo sapiens OX=9606 GN=KLKB1 PE=1 SV=1 | 71.3697 | 638 |
| P04003 | C4BPA_HUMAN | C4BPA | C4b-binding protein alpha chain OS=Homo sapiens OX=9606 GN=C4BPA PE=1 SV=2 | 67.0332 | 597 |
| P04004 | VTNC_HUMAN | VTN | Vitronectin OS=Homo sapiens OX=9606 GN=VTN PE=1 SV=1 | 54.3056 | 478 |
| P04040 | CATA_HUMAN | CAT | Catalase OS=Homo sapiens OX=9606 GN=CAT PE=1 SV=3 | 59.7562 | 527 |
| P04070 | PROC_HUMAN | PROC | Vitamin K-dependent protein C OS=Homo sapiens OX=9606 GN=PROC PE=1 SV=1 | 52.0713 | 461 |
| P04075 | ALDOA_HUMAN | ALDOA | Fructose-bisphosphate aldolase A OS=Homo sapiens OX=9606 GN=ALDOA PE=1 SV=2 | 39.42 | 364 |
| P04083 | ANXA1_HUMAN | ANXA1 | Annexin A1 OS=Homo sapiens OX=9606 GN=ANXA1 PE=1 SV=2 | 38.7143 | 346 |
| P04114 | APOB_HUMAN | APOB | Apolipoprotein B-100 OS=Homo sapiens OX=9606 GN=APOB PE=1 SV=2 | 515.6047 | 4563 |
| P04179 | SODM_HUMAN | SOD2 | Superoxide dismutase [Mn], mitochondrial OS=Homo sapiens OX=9606 GN=SOD2 PE=1 SV=3 | 24.7501 | 222 |
| P04180 | LCAT_HUMAN | LCAT | Phosphatidylcholine-sterol acyltransferase OS=Homo sapiens OX=9606 GN=LCAT PE=1 SV=1 | 49.5779 | 440 |
| P04196 | HRG_HUMAN | HRG | Histidine-rich glycoprotein OS=Homo sapiens OX=9606 GN=HRG PE=1 SV=1 | 59.5783 | 525 |
| P04217 | A1BG_HUMAN | A1BG | Alpha-1B-glycoprotein OS=Homo sapiens OX=9606 GN=A1BG PE=1 SV=4 | 54.2535 | 495 |
| P04264 | K2C1_HUMAN | KRT1 | Keratin, type II cytoskeletal 1 OS=Homo sapiens OX=9606 GN=KRT1 PE=1 SV=6 | 66.0387 | 644 |
| P04275 | VWF_HUMAN | VWF | von Willebrand factor OS=Homo sapiens OX=9606 GN=VWF PE=1 SV=4 | 309.2645 | 2813 |
| P04278 | SHBG_HUMAN | SHBG | Sex hormone-binding globulin OS=Homo sapiens OX=9606 GN=SHBG PE=1 SV=2 | 43.7792 | 402 |
| P04406 | G3P_HUMAN | GAPDH | Glyceraldehyde-3-phosphate dehydrogenase OS=Homo sapiens OX=9606 GN=GAPDH PE=1 SV=3 | 36.0532 | 335 |
| P04430 | KV116_HUMAN | IGKV1-16 | Immunoglobulin kappa variable 1-16 OS=Homo sapiens OX=9606 GN=IGKV1-16 PE=1 SV=2 | 12.6184 | 117 |
| P04439 | HLAA_HUMAN | HLA-A | HLA class I histocompatibility antigen, A alpha chain OS=Homo sapiens OX=9606 GN=HLA-A PE=1 SV=2 | 40.8407 | 365 |
| P04792 | HSPB1_HUMAN | HSPB1 | Heat shock protein beta-1 OS=Homo sapiens OX=9606 GN=HSPB1 PE=1 SV=2 | 22.7825 | 205 |
| P04908 | H2A1B_HUMAN | H2AC4 | Histone H2A type 1-B/E OS=Homo sapiens OX=9606 GN=H2AC4 PE=1 SV=2 | 14.1355 | 130 |
| P05062 | ALDOB_HUMAN | ALDOB | Fructose-bisphosphate aldolase B OS=Homo sapiens OX=9606 GN=ALDOB PE=1 SV=2 | 39.473 | 364 |
| P05067 | A4_HUMAN | APP | Amyloid-beta precursor protein OS=Homo sapiens OX=9606 GN=APP PE=1 SV=3 | 86.9433 | 770 |
| P05090 | APOD_HUMAN | APOD | Apolipoprotein D OS=Homo sapiens OX=9606 GN=APOD PE=1 SV=1 | 21.2756 | 189 |
| P05109 | S10A8_HUMAN | S100A8 | Protein S100-A8 OS=Homo sapiens OX=9606 GN=S100A8 PE=1 SV=1 | 10.8345 | 93 |
| P05141 | ADT2_HUMAN | SLC25A5 | ADP/ATP translocase 2 OS=Homo sapiens OX=9606 GN=SLC25A5 PE=1 SV=7 | 32.8522 | 298 |
| P05154 | IPSP_HUMAN | SERPINA5 | Plasma serine protease inhibitor OS=Homo sapiens OX=9606 GN=SERPINA5 PE=1 SV=3 | 45.6747 | 406 |
| P05155 | IC1_HUMAN | SERPING1 | Plasma protease C1 inhibitor OS=Homo sapiens OX=9606 GN=SERPING1 PE=1 SV=2 | 55.1542 | 500 |
| P05156 | CFAI_HUMAN | CFI | Complement factor I OS=Homo sapiens OX=9606 GN=CFI PE=1 SV=2 | 65.7503 | 583 |
| P05160 | F13B_HUMAN | F13B | Coagulation factor XIII B chain OS=Homo sapiens OX=9606 GN=F13B PE=1 SV=3 | 75.5107 | 661 |
| P05164 | PERM_HUMAN | MPO | Myeloperoxidase OS=Homo sapiens OX=9606 GN=MPO PE=1 SV=1 | 83.8686 | 745 |
| P05362 | ICAM1_HUMAN | ICAM1 | Intercellular adhesion molecule 1 OS=Homo sapiens OX=9606 GN=ICAM1 PE=1 SV=2 | 57.8252 | 532 |
| P05386 | RLA1_HUMAN | RPLP1 | 60S acidic ribosomal protein P1 OS=Homo sapiens OX=9606 GN=RPLP1 PE=1 SV=1 | 11.5139 | 114 |
| P05387 | RLA2_HUMAN | RPLP2 | 60S acidic ribosomal protein P2 OS=Homo sapiens OX=9606 GN=RPLP2 PE=1 SV=1 | 11.6649 | 115 |
| P05452 | TETN_HUMAN | CLEC3B | Tetranectin OS=Homo sapiens OX=9606 GN=CLEC3B PE=1 SV=3 | 22.5368 | 202 |
| P05543 | THBG_HUMAN | SERPINA7 | Thyroxine-binding globulin OS=Homo sapiens OX=9606 GN=SERPINA7 PE=1 SV=2 | 46.3245 | 415 |
| P05546 | HEP2_HUMAN | SERPIND1 | Heparin cofactor 2 OS=Homo sapiens OX=9606 GN=SERPIND1 PE=1 SV=3 | 57.0706 | 499 |
| P05787 | K2C8_HUMAN | KRT8 | Keratin, type II cytoskeletal 8 OS=Homo sapiens OX=9606 GN=KRT8 PE=1 SV=7 | 53.7042 | 483 |
| P06276 | CHLE_HUMAN | BCHE | Cholinesterase OS=Homo sapiens OX=9606 GN=BCHE PE=1 SV=1 | 68.4181 | 602 |
| P06312 | KV401_HUMAN | IGKV4-1 | Immunoglobulin kappa variable 4-1 OS=Homo sapiens OX=9606 GN=IGKV4-1 PE=1 SV=1 | 13.3801 | 121 |
| P06331 | HV434_HUMAN | IGHV4-34 | Immunoglobulin heavy variable 4-34 OS=Homo sapiens OX=9606 GN=IGHV4-34 PE=1 SV=2 | 13.8149 | 123 |
| P06396 | GELS_HUMAN | GSN | Gelsolin OS=Homo sapiens OX=9606 GN=GSN PE=1 SV=1 | 85.6975 | 782 |
| P06576 | ATPB_HUMAN | ATP5F1B | ATP synthase subunit beta, mitochondrial OS=Homo sapiens OX=9606 GN=ATP5F1B PE=1 SV=3 | 56.5599 | 529 |
| P06681 | CO2_HUMAN | C2 | Complement C2 OS=Homo sapiens OX=9606 GN=C2 PE=1 SV=2 | 83.2678 | 752 |
| P06702 | S10A9_HUMAN | S100A9 | Protein S100-A9 OS=Homo sapiens OX=9606 GN=S100A9 PE=1 SV=1 | 13.242 | 114 |
| P06727 | APOA4_HUMAN | APOA4 | Apolipoprotein A-IV OS=Homo sapiens OX=9606 GN=APOA4 PE=1 SV=4 | 45.372 | 396 |
| P06733 | ENOA_HUMAN | ENO1 | Alpha-enolase OS=Homo sapiens OX=9606 GN=ENO1 PE=1 SV=2 | 47.169 | 434 |
| P06744 | G6PI_HUMAN | GPI | Glucose-6-phosphate isomerase OS=Homo sapiens OX=9606 GN=GPI PE=1 SV=4 | 63.1471 | 558 |
| P07195 | LDHB_HUMAN | LDHB | L-lactate dehydrogenase B chain OS=Homo sapiens OX=9606 GN=LDHB PE=1 SV=2 | 36.6385 | 334 |
| P07225 | PROS_HUMAN | PROS1 | Vitamin K-dependent protein S OS=Homo sapiens OX=9606 GN=PROS1 PE=1 SV=1 | 75.1226 | 676 |
| P07333 | CSF1R_HUMAN | CSF1R | Macrophage colony-stimulating factor 1 receptor OS=Homo sapiens OX=9606 GN=CSF1R PE=1 SV=2 | 107.9838 | 972 |
| P07339 | CATD_HUMAN | CTSD | Cathepsin D OS=Homo sapiens OX=9606 GN=CTSD PE=1 SV=1 | 44.5522 | 412 |
| P07355 | ANXA2_HUMAN | ANXA2 | Annexin A2 OS=Homo sapiens OX=9606 GN=ANXA2 PE=1 SV=2 | 38.604 | 339 |
| P07357 | CO8A_HUMAN | C8A | Complement component C8 alpha chain OS=Homo sapiens OX=9606 GN=C8A PE=1 SV=2 | 65.1632 | 584 |
| P07358 | CO8B_HUMAN | C8B | Complement component C8 beta chain OS=Homo sapiens OX=9606 GN=C8B PE=1 SV=3 | 67.0469 | 591 |
| P07359 | GP1BA_HUMAN | GP1BA | Platelet glycoprotein Ib alpha chain OS=Homo sapiens OX=9606 GN=GP1BA PE=1 SV=2 | 71.54 | 652 |
| P07360 | CO8G_HUMAN | C8G | Complement component C8 gamma chain OS=Homo sapiens OX=9606 GN=C8G PE=1 SV=3 | 22.2775 | 202 |
| P07437 | TBB5_HUMAN | TUBB | Tubulin beta chain OS=Homo sapiens OX=9606 GN=TUBB PE=1 SV=2 | 49.6708 | 444 |
| P07737 | PROF1_HUMAN | PFN1 | Profilin-1 OS=Homo sapiens OX=9606 GN=PFN1 PE=1 SV=2 | 15.0542 | 140 |
| P07900 | HS90A_HUMAN | HSP90AA1 | Heat shock protein HSP 90-alpha OS=Homo sapiens OX=9606 GN=HSP90AA1 PE=1 SV=5 | 84.6597 | 732 |
| P07988 | PSPB_HUMAN | SFTPB | Pulmonary surfactant-associated protein B OS=Homo sapiens OX=9606 GN=SFTPB PE=1 SV=3 | 42.1171 | 381 |
| P07996 | TSP1_HUMAN | THBS1 | Thrombospondin-1 OS=Homo sapiens OX=9606 GN=THBS1 PE=1 SV=2 | 129.3827 | 1170 |
| P08185 | CBG_HUMAN | SERPINA6 | Corticosteroid-binding globulin OS=Homo sapiens OX=9606 GN=SERPINA6 PE=1 SV=1 | 45.1409 | 405 |
| P08195 | 4F2_HUMAN | SLC3A2 | 4F2 cell-surface antigen heavy chain OS=Homo sapiens OX=9606 GN=SLC3A2 PE=1 SV=3 | 67.994 | 630 |
| P08238 | HS90B_HUMAN | HSP90AB1 | Heat shock protein HSP 90-beta OS=Homo sapiens OX=9606 GN=HSP90AB1 PE=1 SV=4 | 83.2642 | 724 |
| P08253 | MMP2_HUMAN | MMP2 | 72 kDa type IV collagenase OS=Homo sapiens OX=9606 GN=MMP2 PE=1 SV=2 | 73.8823 | 660 |
| P08294 | SODE_HUMAN | SOD3 | Extracellular superoxide dismutase [Cu-Zn] OS=Homo sapiens OX=9606 GN=SOD3 PE=1 SV=2 | 25.8509 | 240 |
| P08514 | ITA2B_HUMAN | ITGA2B | Integrin alpha-IIb OS=Homo sapiens OX=9606 GN=ITGA2B PE=1 SV=3 | 113.3769 | 1039 |
| P08519 | APOA_HUMAN | LPA | Apolipoprotein(a) OS=Homo sapiens OX=9606 GN=LPA PE=1 SV=1 | 501.3191 | 4548 |
| P08571 | CD14_HUMAN | CD14 | Monocyte differentiation antigen CD14 OS=Homo sapiens OX=9606 GN=CD14 PE=1 SV=2 | 40.0762 | 375 |
| P08603 | CFAH_HUMAN | CFH | Complement factor H OS=Homo sapiens OX=9606 GN=CFH PE=1 SV=4 | 139.0963 | 1231 |
| P08637 | FCG3A_HUMAN | FCGR3A | Low affinity immunoglobulin gamma Fc region receptor III-A OS=Homo sapiens OX=9606 GN=FCGR3A PE=1 SV=2 | 29.0891 | 254 |
| P08670 | VIME_HUMAN | VIM | Vimentin OS=Homo sapiens OX=9606 GN=VIM PE=1 SV=4 | 53.6517 | 466 |
| P08697 | A2AP_HUMAN | SERPINF2 | Alpha-2-antiplasmin OS=Homo sapiens OX=9606 GN=SERPINF2 PE=1 SV=3 | 54.5658 | 491 |
| P08709 | FA7_HUMAN | F7 | Coagulation factor VII OS=Homo sapiens OX=9606 GN=F7 PE=1 SV=1 | 51.5939 | 466 |
| P08729 | K2C7_HUMAN | KRT7 | Keratin, type II cytoskeletal 7 OS=Homo sapiens OX=9606 GN=KRT7 PE=1 SV=5 | 51.3857 | 469 |
| P08779 | K1C16_HUMAN | KRT16 | Keratin, type I cytoskeletal 16 OS=Homo sapiens OX=9606 GN=KRT16 PE=1 SV=4 | 51.2678 | 473 |
| P09172 | DOPO_HUMAN | DBH | Dopamine beta-hydroxylase OS=Homo sapiens OX=9606 GN=DBH PE=1 SV=3 | 69.065 | 617 |
| P09486 | SPRC_HUMAN | SPARC | SPARC OS=Homo sapiens OX=9606 GN=SPARC PE=1 SV=1 | 34.6322 | 303 |
| P09488 | GSTM1_HUMAN | GSTM1 | Glutathione S-transferase Mu 1 OS=Homo sapiens OX=9606 GN=GSTM1 PE=1 SV=3 | 25.7118 | 218 |
| P09871 | C1S_HUMAN | C1S | Complement C1s subcomponent OS=Homo sapiens OX=9606 GN=C1S PE=1 SV=1 | 76.6844 | 688 |
| P0C0L4 | CO4A_HUMAN | C4A | Complement C4-A OS=Homo sapiens OX=9606 GN=C4A PE=1 SV=2 | 192.7855 | 1744 |
| P0C0L5 | CO4B_HUMAN | C4B | Complement C4-B OS=Homo sapiens OX=9606 GN=C4B PE=1 SV=2 | 192.7515 | 1744 |
| P0CG47 | UBB_HUMAN | UBB | Polyubiquitin-B OS=Homo sapiens OX=9606 GN=UBB PE=1 SV=1 | 25.7616 | 229 |
| P0DJI8 | SAA1_HUMAN | SAA1 | Serum amyloid A-1 protein OS=Homo sapiens OX=9606 GN=SAA1 PE=1 SV=1 | 13.532 | 122 |
| P0DME0 | SETLP_HUMAN | SETSIP | Protein SETSIP OS=Homo sapiens OX=9606 GN=SETSIP PE=1 SV=1 | 34.8824 | 302 |
| P0DMV8 | HS71A_HUMAN | HSPA1A | Heat shock 70 kDa protein 1A OS=Homo sapiens OX=9606 GN=HSPA1A PE=1 SV=1 | 70.0522 | 641 |
| P0DOX2 | IGA2_HUMAN |  | Immunoglobulin alpha-2 heavy chain OS=Homo sapiens OX=9606 PE=1 SV=2 | 48.9342 | 455 |
| P0DOX3 | IGD_HUMAN |  | Immunoglobulin delta heavy chain OS=Homo sapiens OX=9606 PE=1 SV=1 | 56.2243 | 512 |
| P0DOX5 | IGG1_HUMAN |  | Immunoglobulin gamma-1 heavy chain OS=Homo sapiens OX=9606 PE=1 SV=2 | 49.3288 | 449 |
| P0DOX6 | IGM_HUMAN |  | Immunoglobulin mu heavy chain OS=Homo sapiens OX=9606 PE=1 SV=2 | 63.4859 | 576 |
| P0DOX7 | IGK_HUMAN |  | Immunoglobulin kappa light chain OS=Homo sapiens OX=9606 PE=1 SV=1 | 23.3791 | 214 |
| P0DOX8 | IGL1_HUMAN |  | Immunoglobulin lambda-1 light chain OS=Homo sapiens OX=9606 PE=1 SV=1 | 22.8302 | 216 |
| P0DOY2 | IGLC2_HUMAN | IGLC2 | Immunoglobulin lambda constant 2 OS=Homo sapiens OX=9606 GN=IGLC2 PE=1 SV=1 | 11.2936 | 106 |
| P0DP01 | HV108_HUMAN | IGHV1-8 | Immunoglobulin heavy variable 1-8 OS=Homo sapiens OX=9606 GN=IGHV1-8 PE=1 SV=1 | 12.9917 | 117 |
| P0DP07 | HV431_HUMAN | IGHV4-31 | Immunoglobulin heavy variable 4-31 OS=Homo sapiens OX=9606 GN=IGHV4-31 PE=3 SV=1 | 13.095 | 118 |
| P10599 | THIO_HUMAN | TXN | Thioredoxin OS=Homo sapiens OX=9606 GN=TXN PE=1 SV=3 | 11.7375 | 105 |
| P10643 | CO7_HUMAN | C7 | Complement component C7 OS=Homo sapiens OX=9606 GN=C7 PE=1 SV=2 | 93.5182 | 843 |
| P10809 | CH60_HUMAN | HSPD1 | 60 kDa heat shock protein, mitochondrial OS=Homo sapiens OX=9606 GN=HSPD1 PE=1 SV=2 | 61.0546 | 573 |
| P10909 | CLUS_HUMAN | CLU | Clusterin OS=Homo sapiens OX=9606 GN=CLU PE=1 SV=1 | 52.4946 | 449 |
| P11021 | BIP_HUMAN | HSPA5 | Endoplasmic reticulum chaperone BiP OS=Homo sapiens OX=9606 GN=HSPA5 PE=1 SV=2 | 72.333 | 654 |
| P11142 | HSP7C_HUMAN | HSPA8 | Heat shock cognate 71 kDa protein OS=Homo sapiens OX=9606 GN=HSPA8 PE=1 SV=1 | 70.8981 | 646 |
| P11226 | MBL2_HUMAN | MBL2 | Mannose-binding protein C OS=Homo sapiens OX=9606 GN=MBL2 PE=1 SV=2 | 26.1435 | 248 |
| P11413 | G6PD_HUMAN | G6PD | Glucose-6-phosphate 1-dehydrogenase OS=Homo sapiens OX=9606 GN=G6PD PE=1 SV=4 | 59.2568 | 515 |
| P11597 | CETP_HUMAN | CETP | Cholesteryl ester transfer protein OS=Homo sapiens OX=9606 GN=CETP PE=1 SV=2 | 54.7562 | 493 |
| P12035 | K2C3_HUMAN | KRT3 | Keratin, type II cytoskeletal 3 OS=Homo sapiens OX=9606 GN=KRT3 PE=1 SV=3 | 64.417 | 628 |
| P12109 | CO6A1_HUMAN | COL6A1 | Collagen alpha-1(VI) chain OS=Homo sapiens OX=9606 GN=COL6A1 PE=1 SV=3 | 108.5294 | 1028 |
| P12111 | CO6A3_HUMAN | COL6A3 | Collagen alpha-3(VI) chain OS=Homo sapiens OX=9606 GN=COL6A3 PE=1 SV=5 | 343.6694 | 3177 |
| P12259 | FA5_HUMAN | F5 | Coagulation factor V OS=Homo sapiens OX=9606 GN=F5 PE=1 SV=4 | 251.7034 | 2224 |
| P12277 | KCRB_HUMAN | CKB | Creatine kinase B-type OS=Homo sapiens OX=9606 GN=CKB PE=1 SV=1 | 42.6443 | 381 |
| P12830 | CADH1_HUMAN | CDH1 | Cadherin-1 OS=Homo sapiens OX=9606 GN=CDH1 PE=1 SV=3 | 97.4562 | 882 |
| P13473 | LAMP2_HUMAN | LAMP2 | Lysosome-associated membrane glycoprotein 2 OS=Homo sapiens OX=9606 GN=LAMP2 PE=1 SV=2 | 44.9607 | 410 |
| P13591 | NCAM1_HUMAN | NCAM1 | Neural cell adhesion molecule 1 OS=Homo sapiens OX=9606 GN=NCAM1 PE=1 SV=3 | 94.5742 | 858 |
| P13598 | ICAM2_HUMAN | ICAM2 | Intercellular adhesion molecule 2 OS=Homo sapiens OX=9606 GN=ICAM2 PE=1 SV=2 | 30.6543 | 275 |
| P13639 | EF2_HUMAN | EEF2 | Elongation factor 2 OS=Homo sapiens OX=9606 GN=EEF2 PE=1 SV=4 | 95.3381 | 858 |
| P13645 | K1C10_HUMAN | KRT10 | Keratin, type I cytoskeletal 10 OS=Homo sapiens OX=9606 GN=KRT10 PE=1 SV=6 | 58.8271 | 584 |
| P13646 | K1C13_HUMAN | KRT13 | Keratin, type I cytoskeletal 13 OS=Homo sapiens OX=9606 GN=KRT13 PE=1 SV=4 | 49.5882 | 458 |
| P13647 | K2C5_HUMAN | KRT5 | Keratin, type II cytoskeletal 5 OS=Homo sapiens OX=9606 GN=KRT5 PE=1 SV=3 | 62.3783 | 590 |
| P13671 | CO6_HUMAN | C6 | Complement component C6 OS=Homo sapiens OX=9606 GN=C6 PE=1 SV=3 | 104.7861 | 934 |
| P13796 | PLSL_HUMAN | LCP1 | Plastin-2 OS=Homo sapiens OX=9606 GN=LCP1 PE=1 SV=6 | 70.2884 | 627 |
| P14151 | LYAM1_HUMAN | SELL | L-selectin OS=Homo sapiens OX=9606 GN=SELL PE=1 SV=2 | 42.1871 | 372 |
| P14543 | NID1_HUMAN | NID1 | Nidogen-1 OS=Homo sapiens OX=9606 GN=NID1 PE=1 SV=3 | 136.377 | 1247 |
| P14618 | KPYM_HUMAN | PKM | Pyruvate kinase PKM OS=Homo sapiens OX=9606 GN=PKM PE=1 SV=4 | 57.9369 | 531 |
| P14625 | ENPL_HUMAN | HSP90B1 | Endoplasmin OS=Homo sapiens OX=9606 GN=HSP90B1 PE=1 SV=1 | 92.4689 | 803 |
| P14780 | MMP9_HUMAN | MMP9 | Matrix metalloproteinase-9 OS=Homo sapiens OX=9606 GN=MMP9 PE=1 SV=3 | 78.4582 | 707 |
| P15144 | AMPN_HUMAN | ANPEP | Aminopeptidase N OS=Homo sapiens OX=9606 GN=ANPEP PE=1 SV=4 | 109.5396 | 967 |
| P15169 | CBPN_HUMAN | CPN1 | Carboxypeptidase N catalytic chain OS=Homo sapiens OX=9606 GN=CPN1 PE=1 SV=1 | 52.2862 | 458 |
| P15259 | PGAM2_HUMAN | PGAM2 | Phosphoglycerate mutase 2 OS=Homo sapiens OX=9606 GN=PGAM2 PE=1 SV=3 | 28.7662 | 253 |
| P15814 | IGLL1_HUMAN | IGLL1 | Immunoglobulin lambda-like polypeptide 1 OS=Homo sapiens OX=9606 GN=IGLL1 PE=1 SV=1 | 22.963 | 213 |
| P15880 | RS2_HUMAN | RPS2 | 40S ribosomal protein S2 OS=Homo sapiens OX=9606 GN=RPS2 PE=1 SV=2 | 31.3244 | 293 |
| P16035 | TIMP2_HUMAN | TIMP2 | Metalloproteinase inhibitor 2 OS=Homo sapiens OX=9606 GN=TIMP2 PE=1 SV=2 | 24.3993 | 220 |
| P16070 | CD44_HUMAN | CD44 | CD44 antigen OS=Homo sapiens OX=9606 GN=CD44 PE=1 SV=3 | 81.5376 | 742 |
| P16930 | FAAA_HUMAN | FAH | Fumarylacetoacetase OS=Homo sapiens OX=9606 GN=FAH PE=1 SV=2 | 46.3743 | 419 |
| P17936 | IBP3_HUMAN | IGFBP3 | Insulin-like growth factor-binding protein 3 OS=Homo sapiens OX=9606 GN=IGFBP3 PE=1 SV=2 | 31.6742 | 291 |
| P18124 | RL7_HUMAN | RPL7 | 60S ribosomal protein L7 OS=Homo sapiens OX=9606 GN=RPL7 PE=1 SV=1 | 29.2258 | 248 |
| P18206 | VINC_HUMAN | VCL | Vinculin OS=Homo sapiens OX=9606 GN=VCL PE=1 SV=4 | 123.7993 | 1134 |
| P18428 | LBP_HUMAN | LBP | Lipopolysaccharide-binding protein OS=Homo sapiens OX=9606 GN=LBP PE=1 SV=3 | 53.3836 | 481 |
| P19320 | VCAM1_HUMAN | VCAM1 | Vascular cell adhesion protein 1 OS=Homo sapiens OX=9606 GN=VCAM1 PE=1 SV=1 | 81.2762 | 739 |
| P19652 | A1AG2_HUMAN | ORM2 | Alpha-1-acid glycoprotein 2 OS=Homo sapiens OX=9606 GN=ORM2 PE=1 SV=2 | 23.6026 | 201 |
| P19823 | ITIH2_HUMAN | ITIH2 | Inter-alpha-trypsin inhibitor heavy chain H2 OS=Homo sapiens OX=9606 GN=ITIH2 PE=1 SV=2 | 106.4635 | 946 |
| P19827 | ITIH1_HUMAN | ITIH1 | Inter-alpha-trypsin inhibitor heavy chain H1 OS=Homo sapiens OX=9606 GN=ITIH1 PE=1 SV=3 | 101.3891 | 911 |
| P20742 | PZP_HUMAN | PZP | Pregnancy zone protein OS=Homo sapiens OX=9606 GN=PZP PE=1 SV=4 | 163.863 | 1482 |
| P20851 | C4BPB_HUMAN | C4BPB | C4b-binding protein beta chain OS=Homo sapiens OX=9606 GN=C4BPB PE=1 SV=1 | 28.3574 | 252 |
| P21333 | FLNA_HUMAN | FLNA | Filamin-A OS=Homo sapiens OX=9606 GN=FLNA PE=1 SV=4 | 280.739 | 2647 |
| P22105 | TENX_HUMAN | TNXB | Tenascin-X OS=Homo sapiens OX=9606 GN=TNXB PE=1 SV=5 | 458.3885 | 4244 |
| P22314 | UBA1_HUMAN | UBA1 | Ubiquitin-like modifier-activating enzyme 1 OS=Homo sapiens OX=9606 GN=UBA1 PE=1 SV=3 | 117.849 | 1058 |
| P22352 | GPX3_HUMAN | GPX3 | Glutathione peroxidase 3 OS=Homo sapiens OX=9606 GN=GPX3 PE=1 SV=2 | 25.5523 | 226 |
| P22792 | CPN2_HUMAN | CPN2 | Carboxypeptidase N subunit 2 OS=Homo sapiens OX=9606 GN=CPN2 PE=1 SV=3 | 60.5567 | 545 |
| P22891 | PROZ_HUMAN | PROZ | Vitamin K-dependent protein Z OS=Homo sapiens OX=9606 GN=PROZ PE=1 SV=2 | 44.7439 | 400 |
| P23083 | HV102_HUMAN | IGHV1-2 | Immunoglobulin heavy variable 1-2 OS=Homo sapiens OX=9606 GN=IGHV1-2 PE=1 SV=2 | 13.0848 | 117 |
| P23142 | FBLN1_HUMAN | FBLN1 | Fibulin-1 OS=Homo sapiens OX=9606 GN=FBLN1 PE=1 SV=4 | 77.2141 | 703 |
| P23470 | PTPRG_HUMAN | PTPRG | Receptor-type tyrosine-protein phosphatase gamma OS=Homo sapiens OX=9606 GN=PTPRG PE=1 SV=4 | 162.0035 | 1445 |
| P23528 | COF1_HUMAN | CFL1 | Cofilin-1 OS=Homo sapiens OX=9606 GN=CFL1 PE=1 SV=3 | 18.5025 | 166 |
| P24158 | PRTN3_HUMAN | PRTN3 | Myeloblastin OS=Homo sapiens OX=9606 GN=PRTN3 PE=1 SV=3 | 27.807 | 256 |
| P24752 | THIL_HUMAN | ACAT1 | Acetyl-CoA acetyltransferase, mitochondrial OS=Homo sapiens OX=9606 GN=ACAT1 PE=1 SV=1 | 45.1996 | 427 |
| P24821 | TENA_HUMAN | TNC | Tenascin OS=Homo sapiens OX=9606 GN=TNC PE=1 SV=3 | 240.8534 | 2201 |
| P25311 | ZA2G_HUMAN | AZGP1 | Zinc-alpha-2-glycoprotein OS=Homo sapiens OX=9606 GN=AZGP1 PE=1 SV=2 | 34.2587 | 298 |
| P25705 | ATPA_HUMAN | ATP5F1A | ATP synthase subunit alpha, mitochondrial OS=Homo sapiens OX=9606 GN=ATP5F1A PE=1 SV=1 | 59.7506 | 553 |
| P26038 | MOES_HUMAN | MSN | Moesin OS=Homo sapiens OX=9606 GN=MSN PE=1 SV=3 | 67.82 | 577 |
| P26641 | EF1G_HUMAN | EEF1G | Elongation factor 1-gamma OS=Homo sapiens OX=9606 GN=EEF1G PE=1 SV=3 | 50.1188 | 437 |
| P26927 | HGFL_HUMAN | MST1 | Hepatocyte growth factor-like protein OS=Homo sapiens OX=9606 GN=MST1 PE=1 SV=2 | 80.3199 | 711 |
| P27169 | PON1_HUMAN | PON1 | Serum paraoxonase/arylesterase 1 OS=Homo sapiens OX=9606 GN=PON1 PE=1 SV=3 | 39.7313 | 355 |
| P27797 | CALR_HUMAN | CALR | Calreticulin OS=Homo sapiens OX=9606 GN=CALR PE=1 SV=1 | 48.1416 | 417 |
| P27918 | PROP_HUMAN | CFP | Properdin OS=Homo sapiens OX=9606 GN=CFP PE=1 SV=2 | 51.2764 | 469 |
| P29622 | KAIN_HUMAN | SERPINA4 | Kallistatin OS=Homo sapiens OX=9606 GN=SERPINA4 PE=1 SV=3 | 48.542 | 427 |
| P30048 | PRDX3_HUMAN | PRDX3 | Thioredoxin-dependent peroxide reductase, mitochondrial OS=Homo sapiens OX=9606 GN=PRDX3 PE=1 SV=3 | 27.6927 | 256 |
| P30050 | RL12_HUMAN | RPL12 | 60S ribosomal protein L12 OS=Homo sapiens OX=9606 GN=RPL12 PE=1 SV=1 | 17.8186 | 165 |
| P30101 | PDIA3_HUMAN | PDIA3 | Protein disulfide-isomerase A3 OS=Homo sapiens OX=9606 GN=PDIA3 PE=1 SV=4 | 56.7824 | 505 |
| P31327 | CPSM_HUMAN | CPS1 | Carbamoyl-phosphate synthase [ammonia], mitochondrial OS=Homo sapiens OX=9606 GN=CPS1 PE=1 SV=2 | 164.9395 | 1500 |
| P31943 | HNRH1_HUMAN | HNRNPH1 | Heterogeneous nuclear ribonucleoprotein H OS=Homo sapiens OX=9606 GN=HNRNPH1 PE=1 SV=4 | 49.2295 | 449 |
| P31946 | 1433B_HUMAN | YWHAB | 14-3-3 protein beta/alpha OS=Homo sapiens OX=9606 GN=YWHAB PE=1 SV=3 | 28.0824 | 246 |
| P32119 | PRDX2_HUMAN | PRDX2 | Peroxiredoxin-2 OS=Homo sapiens OX=9606 GN=PRDX2 PE=1 SV=5 | 21.8919 | 198 |
| P33151 | CADH5_HUMAN | CDH5 | Cadherin-5 OS=Homo sapiens OX=9606 GN=CDH5 PE=1 SV=5 | 87.5284 | 784 |
| P33908 | MA1A1_HUMAN | MAN1A1 | Mannosyl-oligosaccharide 1,2-alpha-mannosidase IA OS=Homo sapiens OX=9606 GN=MAN1A1 PE=1 SV=3 | 72.9685 | 653 |
| P34932 | HSP74_HUMAN | HSPA4 | Heat shock 70 kDa protein 4 OS=Homo sapiens OX=9606 GN=HSPA4 PE=1 SV=4 | 94.3309 | 840 |
| P35030 | TRY3_HUMAN | PRSS3 | Trypsin-3 OS=Homo sapiens OX=9606 GN=PRSS3 PE=1 SV=2 | 32.5288 | 304 |
| P35527 | K1C9_HUMAN | KRT9 | Keratin, type I cytoskeletal 9 OS=Homo sapiens OX=9606 GN=KRT9 PE=1 SV=3 | 62.0643 | 623 |
| P35542 | SAA4_HUMAN | SAA4 | Serum amyloid A-4 protein OS=Homo sapiens OX=9606 GN=SAA4 PE=1 SV=2 | 14.7467 | 130 |
| P35579 | MYH9_HUMAN | MYH9 | Myosin-9 OS=Homo sapiens OX=9606 GN=MYH9 PE=1 SV=4 | 226.5322 | 1960 |
| P35858 | ALS_HUMAN | IGFALS | Insulin-like growth factor-binding protein complex acid labile subunit OS=Homo sapiens OX=9606 GN=IGFALS PE=1 SV=1 | 66.035 | 605 |
| P35908 | K22E_HUMAN | KRT2 | Keratin, type II cytoskeletal 2 epidermal OS=Homo sapiens OX=9606 GN=KRT2 PE=1 SV=2 | 65.4329 | 639 |
| P36578 | RL4_HUMAN | RPL4 | 60S ribosomal protein L4 OS=Homo sapiens OX=9606 GN=RPL4 PE=1 SV=5 | 47.6973 | 427 |
| P36955 | PEDF_HUMAN | SERPINF1 | Pigment epithelium-derived factor OS=Homo sapiens OX=9606 GN=SERPINF1 PE=1 SV=4 | 46.3122 | 418 |
| P37802 | TAGL2_HUMAN | TAGLN2 | Transgelin-2 OS=Homo sapiens OX=9606 GN=TAGLN2 PE=1 SV=3 | 22.3915 | 199 |
| P38646 | GRP75_HUMAN | HSPA9 | Stress-70 protein, mitochondrial OS=Homo sapiens OX=9606 GN=HSPA9 PE=1 SV=2 | 73.6805 | 679 |
| P39060 | COIA1_HUMAN | COL18A1 | Collagen alpha-1(XVIII) chain OS=Homo sapiens OX=9606 GN=COL18A1 PE=1 SV=5 | 178.1877 | 1754 |
| P40197 | GPV_HUMAN | GP5 | Platelet glycoprotein V OS=Homo sapiens OX=9606 GN=GP5 PE=1 SV=1 | 60.9592 | 560 |
| P40926 | MDHM_HUMAN | MDH2 | Malate dehydrogenase, mitochondrial OS=Homo sapiens OX=9606 GN=MDH2 PE=1 SV=3 | 35.5033 | 338 |
| P41222 | PTGDS_HUMAN | PTGDS | Prostaglandin-H2 D-isomerase OS=Homo sapiens OX=9606 GN=PTGDS PE=1 SV=1 | 21.0288 | 190 |
| P41250 | GARS_HUMAN | GARS1 | Glycine--tRNA ligase OS=Homo sapiens OX=9606 GN=GARS1 PE=1 SV=3 | 83.1656 | 739 |
| P43121 | MUC18_HUMAN | MCAM | Cell surface glycoprotein MUC18 OS=Homo sapiens OX=9606 GN=MCAM PE=1 SV=2 | 71.6074 | 646 |
| P43243 | MATR3_HUMAN | MATR3 | Matrin-3 OS=Homo sapiens OX=9606 GN=MATR3 PE=1 SV=2 | 94.6232 | 847 |
| P43251 | BTD_HUMAN | BTD | Biotinidase OS=Homo sapiens OX=9606 GN=BTD PE=1 SV=2 | 61.1329 | 543 |
| P43652 | AFAM_HUMAN | AFM | Afamin OS=Homo sapiens OX=9606 GN=AFM PE=1 SV=1 | 69.0691 | 599 |
| P46783 | RS10_HUMAN | RPS10 | 40S ribosomal protein S10 OS=Homo sapiens OX=9606 GN=RPS10 PE=1 SV=1 | 18.8978 | 165 |
| P48740 | MASP1_HUMAN | MASP1 | Mannan-binding lectin serine protease 1 OS=Homo sapiens OX=9606 GN=MASP1 PE=1 SV=3 | 79.2467 | 699 |
| P49411 | EFTU_HUMAN | TUFM | Elongation factor Tu, mitochondrial OS=Homo sapiens OX=9606 GN=TUFM PE=1 SV=2 | 49.5415 | 452 |
| P49747 | COMP_HUMAN | COMP | Cartilage oligomeric matrix protein OS=Homo sapiens OX=9606 GN=COMP PE=1 SV=2 | 82.8605 | 757 |
| P49908 | SEPP1_HUMAN | SELENOP | Selenoprotein P OS=Homo sapiens OX=9606 GN=SELENOP PE=1 SV=3 | 43.1736 | 381 |
| P49913 | CAMP_HUMAN | CAMP | Cathelicidin antimicrobial peptide OS=Homo sapiens OX=9606 GN=CAMP PE=1 SV=1 | 19.3014 | 170 |
| P50991 | TCPD_HUMAN | CCT4 | T-complex protein 1 subunit delta OS=Homo sapiens OX=9606 GN=CCT4 PE=1 SV=4 | 57.9242 | 539 |
| P51884 | LUM_HUMAN | LUM | Lumican OS=Homo sapiens OX=9606 GN=LUM PE=1 SV=2 | 38.429 | 338 |
| P52907 | CAZA1_HUMAN | CAPZA1 | F-actin-capping protein subunit alpha-1 OS=Homo sapiens OX=9606 GN=CAPZA1 PE=1 SV=3 | 32.9228 | 286 |
| P53621 | COPA_HUMAN | COPA | Coatomer subunit alpha OS=Homo sapiens OX=9606 GN=COPA PE=1 SV=2 | 138.3458 | 1224 |
| P55056 | APOC4_HUMAN | APOC4 | Apolipoprotein C-IV OS=Homo sapiens OX=9606 GN=APOC4 PE=1 SV=1 | 14.5531 | 127 |
| P55058 | PLTP_HUMAN | PLTP | Phospholipid transfer protein OS=Homo sapiens OX=9606 GN=PLTP PE=1 SV=1 | 54.7394 | 493 |
| P55072 | TERA_HUMAN | VCP | Transitional endoplasmic reticulum ATPase OS=Homo sapiens OX=9606 GN=VCP PE=1 SV=4 | 89.3218 | 806 |
| P55103 | INHBC_HUMAN | INHBC | Inhibin beta C chain OS=Homo sapiens OX=9606 GN=INHBC PE=2 SV=1 | 38.2379 | 352 |
| P55209 | NP1L1_HUMAN | NAP1L1 | Nucleosome assembly protein 1-like 1 OS=Homo sapiens OX=9606 GN=NAP1L1 PE=1 SV=1 | 45.3742 | 391 |
| P55290 | CAD13_HUMAN | CDH13 | Cadherin-13 OS=Homo sapiens OX=9606 GN=CDH13 PE=1 SV=1 | 78.2869 | 713 |
| P58335 | ANTR2_HUMAN | ANTXR2 | Anthrax toxin receptor 2 OS=Homo sapiens OX=9606 GN=ANTXR2 PE=1 SV=5 | 53.6662 | 489 |
| P60174 | TPIS_HUMAN | TPI1 | Triosephosphate isomerase OS=Homo sapiens OX=9606 GN=TPI1 PE=1 SV=4 | 26.6695 | 249 |
| P60709 | ACTB_HUMAN | ACTB | Actin, cytoplasmic 1 OS=Homo sapiens OX=9606 GN=ACTB PE=1 SV=1 | 41.7367 | 375 |
| P60842 | IF4A1_HUMAN | EIF4A1 | Eukaryotic initiation factor 4A-I OS=Homo sapiens OX=9606 GN=EIF4A1 PE=1 SV=1 | 46.1539 | 406 |
| P60953 | CDC42_HUMAN | CDC42 | Cell division control protein 42 homolog OS=Homo sapiens OX=9606 GN=CDC42 PE=1 SV=2 | 21.2586 | 191 |
| P61204 | ARF3_HUMAN | ARF3 | ADP-ribosylation factor 3 OS=Homo sapiens OX=9606 GN=ARF3 PE=1 SV=2 | 20.6008 | 181 |
| P61224 | RAP1B_HUMAN | RAP1B | Ras-related protein Rap-1b OS=Homo sapiens OX=9606 GN=RAP1B PE=1 SV=1 | 20.8248 | 184 |
| P61626 | LYSC_HUMAN | LYZ | Lysozyme C OS=Homo sapiens OX=9606 GN=LYZ PE=1 SV=1 | 16.537 | 148 |
| P61769 | B2MG_HUMAN | B2M | Beta-2-microglobulin OS=Homo sapiens OX=9606 GN=B2M PE=1 SV=1 | 13.7146 | 119 |
| P61978 | HNRPK_HUMAN | HNRNPK | Heterogeneous nuclear ribonucleoprotein K OS=Homo sapiens OX=9606 GN=HNRNPK PE=1 SV=1 | 50.9763 | 463 |
| P62081 | RS7_HUMAN | RPS7 | 40S ribosomal protein S7 OS=Homo sapiens OX=9606 GN=RPS7 PE=1 SV=1 | 22.1269 | 194 |
| P62241 | RS8_HUMAN | RPS8 | 40S ribosomal protein S8 OS=Homo sapiens OX=9606 GN=RPS8 PE=1 SV=2 | 24.2052 | 208 |
| P62258 | 1433E_HUMAN | YWHAE | 14-3-3 protein epsilon OS=Homo sapiens OX=9606 GN=YWHAE PE=1 SV=1 | 29.1739 | 255 |
| P62277 | RS13_HUMAN | RPS13 | 40S ribosomal protein S13 OS=Homo sapiens OX=9606 GN=RPS13 PE=1 SV=2 | 17.2223 | 151 |
| P62701 | RS4X_HUMAN | RPS4X | 40S ribosomal protein S4, X isoform OS=Homo sapiens OX=9606 GN=RPS4X PE=1 SV=2 | 29.5977 | 263 |
| P62736 | ACTA_HUMAN | ACTA2 | Actin, aortic smooth muscle OS=Homo sapiens OX=9606 GN=ACTA2 PE=1 SV=1 | 42.009 | 377 |
| P62805 | H4_HUMAN | H4C1 | Histone H4 OS=Homo sapiens OX=9606 GN=H4C1 PE=1 SV=2 | 11.3673 | 103 |
| P62820 | RAB1A_HUMAN | RAB1A | Ras-related protein Rab-1A OS=Homo sapiens OX=9606 GN=RAB1A PE=1 SV=3 | 22.6778 | 205 |
| P62826 | RAN_HUMAN | RAN | GTP-binding nuclear protein Ran OS=Homo sapiens OX=9606 GN=RAN PE=1 SV=3 | 24.4231 | 216 |
| P62829 | RL23_HUMAN | RPL23 | 60S ribosomal protein L23 OS=Homo sapiens OX=9606 GN=RPL23 PE=1 SV=1 | 14.8654 | 140 |
| P62857 | RS28_HUMAN | RPS28 | 40S ribosomal protein S28 OS=Homo sapiens OX=9606 GN=RPS28 PE=1 SV=1 | 7.841 | 69 |
| P62888 | RL30_HUMAN | RPL30 | 60S ribosomal protein L30 OS=Homo sapiens OX=9606 GN=RPL30 PE=1 SV=2 | 12.7841 | 115 |
| P62899 | RL31_HUMAN | RPL31 | 60S ribosomal protein L31 OS=Homo sapiens OX=9606 GN=RPL31 PE=1 SV=1 | 14.4629 | 125 |
| P62937 | PPIA_HUMAN | PPIA | Peptidyl-prolyl cis-trans isomerase A OS=Homo sapiens OX=9606 GN=PPIA PE=1 SV=2 | 18.0125 | 165 |
| P63104 | 1433Z_HUMAN | YWHAZ | 14-3-3 protein zeta/delta OS=Homo sapiens OX=9606 GN=YWHAZ PE=1 SV=1 | 27.7451 | 245 |
| P67936 | TPM4_HUMAN | TPM4 | Tropomyosin alpha-4 chain OS=Homo sapiens OX=9606 GN=TPM4 PE=1 SV=3 | 28.5218 | 248 |
| P68104 | EF1A1_HUMAN | EEF1A1 | Elongation factor 1-alpha 1 OS=Homo sapiens OX=9606 GN=EEF1A1 PE=1 SV=1 | 50.1409 | 462 |
| P68363 | TBA1B_HUMAN | TUBA1B | Tubulin alpha-1B chain OS=Homo sapiens OX=9606 GN=TUBA1B PE=1 SV=1 | 50.1516 | 451 |
| P68366 | TBA4A_HUMAN | TUBA4A | Tubulin alpha-4A chain OS=Homo sapiens OX=9606 GN=TUBA4A PE=1 SV=1 | 49.9244 | 448 |
| P68371 | TBB4B_HUMAN | TUBB4B | Tubulin beta-4B chain OS=Homo sapiens OX=9606 GN=TUBB4B PE=1 SV=1 | 49.831 | 445 |
| P68871 | HBB_HUMAN | HBB | Hemoglobin subunit beta OS=Homo sapiens OX=9606 GN=HBB PE=1 SV=2 | 15.9984 | 147 |
| P69891 | HBG1_HUMAN | HBG1 | Hemoglobin subunit gamma-1 OS=Homo sapiens OX=9606 GN=HBG1 PE=1 SV=2 | 16.1405 | 147 |
| P69905 | HBA_HUMAN | HBA1 | Hemoglobin subunit alpha OS=Homo sapiens OX=9606 GN=HBA1 PE=1 SV=2 | 15.2576 | 142 |
| P80108 | PHLD_HUMAN | GPLD1 | Phosphatidylinositol-glycan-specific phospholipase D OS=Homo sapiens OX=9606 GN=GPLD1 PE=1 SV=3 | 92.3365 | 840 |
| P80188 | NGAL_HUMAN | LCN2 | Neutrophil gelatinase-associated lipocalin OS=Homo sapiens OX=9606 GN=LCN2 PE=1 SV=2 | 22.5881 | 198 |
| P80748 | LV321_HUMAN | IGLV3-21 | Immunoglobulin lambda variable 3-21 OS=Homo sapiens OX=9606 GN=IGLV3-21 PE=1 SV=2 | 12.4457 | 117 |
| P98160 | PGBM_HUMAN | HSPG2 | Basement membrane-specific heparan sulfate proteoglycan core protein OS=Homo sapiens OX=9606 GN=HSPG2 PE=1 SV=4 | 468.83 | 4391 |
| Q00535 | CDK5_HUMAN | CDK5 | Cyclin-dependent-like kinase 5 OS=Homo sapiens OX=9606 GN=CDK5 PE=1 SV=3 | 33.3045 | 292 |
| Q00610 | CLH1_HUMAN | CLTC | Clathrin heavy chain 1 OS=Homo sapiens OX=9606 GN=CLTC PE=1 SV=5 | 191.6146 | 1675 |
| Q00839 | HNRPU_HUMAN | HNRNPU | Heterogeneous nuclear ribonucleoprotein U OS=Homo sapiens OX=9606 GN=HNRNPU PE=1 SV=6 | 90.5844 | 825 |
| Q01459 | DIAC_HUMAN | CTBS | Di-N-acetylchitobiase OS=Homo sapiens OX=9606 GN=CTBS PE=1 SV=1 | 43.7598 | 385 |
| Q01518 | CAP1_HUMAN | CAP1 | Adenylyl cyclase-associated protein 1 OS=Homo sapiens OX=9606 GN=CAP1 PE=1 SV=5 | 51.9015 | 475 |
| Q01973 | ROR1_HUMAN | ROR1 | Inactive tyrosine-protein kinase transmembrane receptor ROR1 OS=Homo sapiens OX=9606 GN=ROR1 PE=1 SV=2 | 104.2826 | 937 |
| Q02878 | RL6_HUMAN | RPL6 | 60S ribosomal protein L6 OS=Homo sapiens OX=9606 GN=RPL6 PE=1 SV=3 | 32.7279 | 288 |
| Q03591 | FHR1_HUMAN | CFHR1 | Complement factor H-related protein 1 OS=Homo sapiens OX=9606 GN=CFHR1 PE=1 SV=2 | 37.6506 | 330 |
| Q04756 | HGFA_HUMAN | HGFAC | Hepatocyte growth factor activator OS=Homo sapiens OX=9606 GN=HGFAC PE=1 SV=1 | 70.6818 | 655 |
| Q06033 | ITIH3_HUMAN | ITIH3 | Inter-alpha-trypsin inhibitor heavy chain H3 OS=Homo sapiens OX=9606 GN=ITIH3 PE=1 SV=2 | 99.8492 | 890 |
| Q08380 | LG3BP_HUMAN | LGALS3BP | Galectin-3-binding protein OS=Homo sapiens OX=9606 GN=LGALS3BP PE=1 SV=1 | 65.331 | 585 |
| Q08J23 | NSUN2_HUMAN | NSUN2 | RNA cytosine C(5)-methyltransferase NSUN2 OS=Homo sapiens OX=9606 GN=NSUN2 PE=1 SV=2 | 86.4707 | 767 |
| Q09666 | AHNK_HUMAN | AHNAK | Neuroblast differentiation-associated protein AHNAK OS=Homo sapiens OX=9606 GN=AHNAK PE=1 SV=2 | 629.1012 | 5890 |
| Q12805 | FBLN3_HUMAN | EFEMP1 | EGF-containing fibulin-like extracellular matrix protein 1 OS=Homo sapiens OX=9606 GN=EFEMP1 PE=1 SV=2 | 54.6406 | 493 |
| Q12860 | CNTN1_HUMAN | CNTN1 | Contactin-1 OS=Homo sapiens OX=9606 GN=CNTN1 PE=1 SV=1 | 113.3204 | 1018 |
| Q12907 | LMAN2_HUMAN | LMAN2 | Vesicular integral-membrane protein VIP36 OS=Homo sapiens OX=9606 GN=LMAN2 PE=1 SV=1 | 40.2287 | 356 |
| Q12913 | PTPRJ_HUMAN | PTPRJ | Receptor-type tyrosine-protein phosphatase eta OS=Homo sapiens OX=9606 GN=PTPRJ PE=1 SV=3 | 145.9415 | 1337 |
| Q12931 | TRAP1_HUMAN | TRAP1 | Heat shock protein 75 kDa, mitochondrial OS=Homo sapiens OX=9606 GN=TRAP1 PE=1 SV=3 | 80.11 | 704 |
| Q13093 | PAFA_HUMAN | PLA2G7 | Platelet-activating factor acetylhydrolase OS=Homo sapiens OX=9606 GN=PLA2G7 PE=1 SV=1 | 50.0774 | 441 |
| Q13103 | SPP24_HUMAN | SPP2 | Secreted phosphoprotein 24 OS=Homo sapiens OX=9606 GN=SPP2 PE=1 SV=1 | 24.3377 | 211 |
| Q13201 | MMRN1_HUMAN | MMRN1 | Multimerin-1 OS=Homo sapiens OX=9606 GN=MMRN1 PE=1 SV=3 | 138.1102 | 1228 |
| Q13263 | TIF1B_HUMAN | TRIM28 | Transcription intermediary factor 1-beta OS=Homo sapiens OX=9606 GN=TRIM28 PE=1 SV=5 | 88.5497 | 835 |
| Q13740 | CD166_HUMAN | ALCAM | CD166 antigen OS=Homo sapiens OX=9606 GN=ALCAM PE=1 SV=2 | 65.1023 | 583 |
| Q13790 | APOF_HUMAN | APOF | Apolipoprotein F OS=Homo sapiens OX=9606 GN=APOF PE=1 SV=2 | 35.3995 | 326 |
| Q14126 | DSG2_HUMAN | DSG2 | Desmoglein-2 OS=Homo sapiens OX=9606 GN=DSG2 PE=1 SV=2 | 122.2939 | 1118 |
| Q14520 | HABP2_HUMAN | HABP2 | Hyaluronan-binding protein 2 OS=Homo sapiens OX=9606 GN=HABP2 PE=1 SV=1 | 62.6717 | 560 |
| Q14623 | IHH_HUMAN | IHH | Indian hedgehog protein OS=Homo sapiens OX=9606 GN=IHH PE=1 SV=4 | 45.2507 | 411 |
| Q14624 | ITIH4_HUMAN | ITIH4 | Inter-alpha-trypsin inhibitor heavy chain H4 OS=Homo sapiens OX=9606 GN=ITIH4 PE=1 SV=4 | 103.3574 | 930 |
| Q14766 | LTBP1_HUMAN | LTBP1 | Latent-transforming growth factor beta-binding protein 1 OS=Homo sapiens OX=9606 GN=LTBP1 PE=1 SV=4 | 186.7961 | 1721 |
| Q14974 | IMB1_HUMAN | KPNB1 | Importin subunit beta-1 OS=Homo sapiens OX=9606 GN=KPNB1 PE=1 SV=2 | 97.1702 | 876 |
| Q14CM0 | FRPD4_HUMAN | FRMPD4 | FERM and PDZ domain-containing protein 4 OS=Homo sapiens OX=9606 GN=FRMPD4 PE=1 SV=1 | 144.3794 | 1322 |
| Q15063 | POSTN_HUMAN | POSTN | Periostin OS=Homo sapiens OX=9606 GN=POSTN PE=1 SV=2 | 93.314 | 836 |
| Q15084 | PDIA6_HUMAN | PDIA6 | Protein disulfide-isomerase A6 OS=Homo sapiens OX=9606 GN=PDIA6 PE=1 SV=1 | 48.1213 | 440 |
| Q15113 | PCOC1_HUMAN | PCOLCE | Procollagen C-endopeptidase enhancer 1 OS=Homo sapiens OX=9606 GN=PCOLCE PE=1 SV=2 | 47.9725 | 449 |
| Q15166 | PON3_HUMAN | PON3 | Serum paraoxonase/lactonase 3 OS=Homo sapiens OX=9606 GN=PON3 PE=1 SV=3 | 39.6075 | 354 |
| Q15185 | TEBP_HUMAN | PTGES3 | Prostaglandin E synthase 3 OS=Homo sapiens OX=9606 GN=PTGES3 PE=1 SV=1 | 18.6974 | 160 |
| Q15366 | PCBP2_HUMAN | PCBP2 | Poly(rC)-binding protein 2 OS=Homo sapiens OX=9606 GN=PCBP2 PE=1 SV=1 | 38.5801 | 365 |
| Q15485 | FCN2_HUMAN | FCN2 | Ficolin-2 OS=Homo sapiens OX=9606 GN=FCN2 PE=1 SV=2 | 34.0011 | 313 |
| Q15582 | BGH3_HUMAN | TGFBI | Transforming growth factor-beta-induced protein ig-h3 OS=Homo sapiens OX=9606 GN=TGFBI PE=1 SV=1 | 74.6809 | 683 |
| Q15758 | AAAT_HUMAN | SLC1A5 | Neutral amino acid transporter B(0) OS=Homo sapiens OX=9606 GN=SLC1A5 PE=1 SV=2 | 56.5983 | 541 |
| Q15828 | CYTM_HUMAN | CST6 | Cystatin-M OS=Homo sapiens OX=9606 GN=CST6 PE=1 SV=1 | 16.5111 | 149 |
| Q15848 | ADIPO_HUMAN | ADIPOQ | Adiponectin OS=Homo sapiens OX=9606 GN=ADIPOQ PE=1 SV=1 | 26.4136 | 244 |
| Q16610 | ECM1_HUMAN | ECM1 | Extracellular matrix protein 1 OS=Homo sapiens OX=9606 GN=ECM1 PE=1 SV=2 | 60.6741 | 540 |
| Q16706 | MA2A1_HUMAN | MAN2A1 | Alpha-mannosidase 2 OS=Homo sapiens OX=9606 GN=MAN2A1 PE=1 SV=2 | 131.1406 | 1144 |
| Q16853 | AOC3_HUMAN | AOC3 | Membrane primary amine oxidase OS=Homo sapiens OX=9606 GN=AOC3 PE=1 SV=3 | 84.6219 | 763 |
| Q17RS7 | GEN_HUMAN | GEN1 | Flap endonuclease GEN homolog 1 OS=Homo sapiens OX=9606 GN=GEN1 PE=1 SV=2 | 102.8845 | 908 |
| Q4G0P3 | HYDIN_HUMAN | HYDIN | Hydrocephalus-inducing protein homolog OS=Homo sapiens OX=9606 GN=HYDIN PE=1 SV=3 | 575.8921 | 5121 |
| Q53FA7 | QORX_HUMAN | TP53I3 | Quinone oxidoreductase PIG3 OS=Homo sapiens OX=9606 GN=TP53I3 PE=1 SV=2 | 35.5362 | 332 |
| Q562R1 | ACTBL_HUMAN | ACTBL2 | Beta-actin-like protein 2 OS=Homo sapiens OX=9606 GN=ACTBL2 PE=1 SV=2 | 42.0032 | 376 |
| Q5JU85 | IQEC2_HUMAN | IQSEC2 | IQ motif and SEC7 domain-containing protein 2 OS=Homo sapiens OX=9606 GN=IQSEC2 PE=1 SV=2 | 162.7836 | 1488 |
| Q6EMK4 | VASN_HUMAN | VASN | Vasorin OS=Homo sapiens OX=9606 GN=VASN PE=1 SV=1 | 71.7127 | 673 |
| Q6UX71 | PXDC2_HUMAN | PLXDC2 | Plexin domain-containing protein 2 OS=Homo sapiens OX=9606 GN=PLXDC2 PE=1 SV=1 | 59.5832 | 529 |
| Q6UXB8 | PI16_HUMAN | PI16 | Peptidase inhibitor 16 OS=Homo sapiens OX=9606 GN=PI16 PE=1 SV=1 | 49.4711 | 463 |
| Q6V1P9 | PCD23_HUMAN | DCHS2 | Protocadherin-23 OS=Homo sapiens OX=9606 GN=DCHS2 PE=2 SV=2 | 370.2284 | 3371 |
| Q76LX8 | ATS13_HUMAN | ADAMTS13 | A disintegrin and metalloproteinase with thrombospondin motifs 13 OS=Homo sapiens OX=9606 GN=ADAMTS13 PE=1 SV=1 | 153.6045 | 1427 |
| Q7Z7G0 | TARSH_HUMAN | ABI3BP | Target of Nesh-SH3 OS=Homo sapiens OX=9606 GN=ABI3BP PE=1 SV=2 | 117.8951 | 1068 |
| Q86U17 | SPA11_HUMAN | SERPINA11 | Serpin A11 OS=Homo sapiens OX=9606 GN=SERPINA11 PE=2 SV=2 | 46.9892 | 422 |
| Q86UD1 | OAF_HUMAN | OAF | Out at first protein homolog OS=Homo sapiens OX=9606 GN=OAF PE=2 SV=1 | 30.6883 | 273 |
| Q86UX7 | URP2_HUMAN | FERMT3 | Fermitin family homolog 3 OS=Homo sapiens OX=9606 GN=FERMT3 PE=1 SV=1 | 75.9528 | 667 |
| Q86VB7 | C163A_HUMAN | CD163 | Scavenger receptor cysteine-rich type 1 protein M130 OS=Homo sapiens OX=9606 GN=CD163 PE=1 SV=2 | 125.4506 | 1156 |
| Q86VP6 | CAND1_HUMAN | CAND1 | Cullin-associated NEDD8-dissociated protein 1 OS=Homo sapiens OX=9606 GN=CAND1 PE=1 SV=2 | 136.3757 | 1230 |
| Q8IUX8 | EGFL6_HUMAN | EGFL6 | Epidermal growth factor-like protein 6 OS=Homo sapiens OX=9606 GN=EGFL6 PE=1 SV=1 | 61.3171 | 553 |
| Q8IVM8 | S22A9_HUMAN | SLC22A9 | Solute carrier family 22 member 9 OS=Homo sapiens OX=9606 GN=SLC22A9 PE=1 SV=1 | 62.1692 | 553 |
| Q8NBP7 | PCSK9_HUMAN | PCSK9 | Proprotein convertase subtilisin/kexin type 9 OS=Homo sapiens OX=9606 GN=PCSK9 PE=1 SV=3 | 74.2862 | 692 |
| Q8NC51 | PAIRB_HUMAN | SERBP1 | Plasminogen activator inhibitor 1 RNA-binding protein OS=Homo sapiens OX=9606 GN=SERBP1 PE=1 SV=2 | 44.9654 | 408 |
| Q8ND04 | SMG8_HUMAN | SMG8 | Protein SMG8 OS=Homo sapiens OX=9606 GN=SMG8 PE=1 SV=1 | 109.6837 | 991 |
| Q8TDL5 | BPIB1_HUMAN | BPIFB1 | BPI fold-containing family B member 1 OS=Homo sapiens OX=9606 GN=BPIFB1 PE=1 SV=1 | 52.4415 | 484 |
| Q8WWZ8 | OIT3_HUMAN | OIT3 | Oncoprotein-induced transcript 3 protein OS=Homo sapiens OX=9606 GN=OIT3 PE=1 SV=2 | 60.0218 | 545 |
| Q92496 | FHR4_HUMAN | CFHR4 | Complement factor H-related protein 4 OS=Homo sapiens OX=9606 GN=CFHR4 PE=1 SV=3 | 65.3508 | 578 |
| Q92820 | GGH_HUMAN | GGH | Gamma-glutamyl hydrolase OS=Homo sapiens OX=9606 GN=GGH PE=1 SV=2 | 35.9644 | 318 |
| Q92954 | PRG4_HUMAN | PRG4 | Proteoglycan 4 OS=Homo sapiens OX=9606 GN=PRG4 PE=1 SV=3 | 151.0611 | 1404 |
| Q96IY4 | CBPB2_HUMAN | CPB2 | Carboxypeptidase B2 OS=Homo sapiens OX=9606 GN=CPB2 PE=1 SV=2 | 48.4243 | 423 |
| Q96KN2 | CNDP1_HUMAN | CNDP1 | Beta-Ala-His dipeptidase OS=Homo sapiens OX=9606 GN=CNDP1 PE=1 SV=4 | 56.7061 | 507 |
| Q96PD5 | PGRP2_HUMAN | PGLYRP2 | N-acetylmuramoyl-L-alanine amidase OS=Homo sapiens OX=9606 GN=PGLYRP2 PE=1 SV=1 | 62.217 | 576 |
| Q99459 | CDC5L_HUMAN | CDC5L | Cell division cycle 5-like protein OS=Homo sapiens OX=9606 GN=CDC5L PE=1 SV=2 | 92.2506 | 802 |
| Q99592 | ZBT18_HUMAN | ZBTB18 | Zinc finger and BTB domain-containing protein 18 OS=Homo sapiens OX=9606 GN=ZBTB18 PE=1 SV=1 | 58.3544 | 522 |
| Q99623 | PHB2_HUMAN | PHB2 | Prohibitin-2 OS=Homo sapiens OX=9606 GN=PHB2 PE=1 SV=2 | 33.2964 | 299 |
| Q99784 | NOE1_HUMAN | OLFM1 | Noelin OS=Homo sapiens OX=9606 GN=OLFM1 PE=1 SV=4 | 55.3426 | 485 |
| Q99969 | RARR2_HUMAN | RARRES2 | Retinoic acid receptor responder protein 2 OS=Homo sapiens OX=9606 GN=RARRES2 PE=1 SV=1 | 18.6176 | 163 |
| Q9BQG0 | MBB1A_HUMAN | MYBBP1A | Myb-binding protein 1A OS=Homo sapiens OX=9606 GN=MYBBP1A PE=1 SV=2 | 148.8546 | 1328 |
| Q9BTY2 | FUCO2_HUMAN | FUCA2 | Plasma alpha-L-fucosidase OS=Homo sapiens OX=9606 GN=FUCA2 PE=1 SV=2 | 54.0667 | 467 |
| Q9BWP8 | COL11_HUMAN | COLEC11 | Collectin-11 OS=Homo sapiens OX=9606 GN=COLEC11 PE=1 SV=1 | 28.6654 | 271 |
| Q9BXR6 | FHR5_HUMAN | CFHR5 | Complement factor H-related protein 5 OS=Homo sapiens OX=9606 GN=CFHR5 PE=1 SV=1 | 64.4194 | 569 |
| Q9BZ67 | FRMD8_HUMAN | FRMD8 | FERM domain-containing protein 8 OS=Homo sapiens OX=9606 GN=FRMD8 PE=1 SV=1 | 51.218 | 464 |
| Q9H4G4 | GAPR1_HUMAN | GLIPR2 | Golgi-associated plant pathogenesis-related protein 1 OS=Homo sapiens OX=9606 GN=GLIPR2 PE=1 SV=3 | 17.2183 | 154 |
| Q9H706 | GARE1_HUMAN | GAREM1 | GRB2-associated and regulator of MAPK protein 1 OS=Homo sapiens OX=9606 GN=GAREM1 PE=1 SV=2 | 97.1861 | 876 |
| Q9H8L6 | MMRN2_HUMAN | MMRN2 | Multimerin-2 OS=Homo sapiens OX=9606 GN=MMRN2 PE=1 SV=2 | 104.4086 | 949 |
| Q9HDC9 | APMAP_HUMAN | APMAP | Adipocyte plasma membrane-associated protein OS=Homo sapiens OX=9606 GN=APMAP PE=1 SV=2 | 46.4804 | 416 |
| Q9NPH3 | IL1AP_HUMAN | IL1RAP | Interleukin-1 receptor accessory protein OS=Homo sapiens OX=9606 GN=IL1RAP PE=1 SV=2 | 65.4184 | 570 |
| Q9NQ79 | CRAC1_HUMAN | CRTAC1 | Cartilage acidic protein 1 OS=Homo sapiens OX=9606 GN=CRTAC1 PE=1 SV=2 | 71.4207 | 661 |
| Q9NR30 | DDX21_HUMAN | DDX21 | Nucleolar RNA helicase 2 OS=Homo sapiens OX=9606 GN=DDX21 PE=1 SV=5 | 87.3444 | 783 |
| Q9NZP8 | C1RL_HUMAN | C1RL | Complement C1r subcomponent-like protein OS=Homo sapiens OX=9606 GN=C1RL PE=1 SV=2 | 53.4983 | 487 |
| Q9P203 | BTBD7_HUMAN | BTBD7 | BTB/POZ domain-containing protein 7 OS=Homo sapiens OX=9606 GN=BTBD7 PE=1 SV=3 | 126.3681 | 1132 |
| Q9UBC5 | MYO1A_HUMAN | MYO1A | Unconventional myosin-Ia OS=Homo sapiens OX=9606 GN=MYO1A PE=1 SV=1 | 118.4006 | 1043 |
| Q9UBI6 | GBG12_HUMAN | GNG12 | Guanine nucleotide-binding protein G(I)/G(S)/G(O) subunit gamma-12 OS=Homo sapiens OX=9606 GN=GNG12 PE=1 SV=3 | 8.0062 | 72 |
| Q9UBP4 | DKK3_HUMAN | DKK3 | Dickkopf-related protein 3 OS=Homo sapiens OX=9606 GN=DKK3 PE=1 SV=2 | 38.3903 | 350 |
| Q9UGM5 | FETUB_HUMAN | FETUB | Fetuin-B OS=Homo sapiens OX=9606 GN=FETUB PE=1 SV=2 | 42.0549 | 382 |
| Q9UHG3 | PCYOX_HUMAN | PCYOX1 | Prenylcysteine oxidase 1 OS=Homo sapiens OX=9606 GN=PCYOX1 PE=1 SV=3 | 56.6401 | 505 |
| Q9UK55 | ZPI_HUMAN | SERPINA10 | Protein Z-dependent protease inhibitor OS=Homo sapiens OX=9606 GN=SERPINA10 PE=1 SV=1 | 50.707 | 444 |
| Q9UNW1 | MINP1_HUMAN | MINPP1 | Multiple inositol polyphosphate phosphatase 1 OS=Homo sapiens OX=9606 GN=MINPP1 PE=1 SV=1 | 55.0512 | 487 |
| Q9Y295 | DRG1_HUMAN | DRG1 | Developmentally-regulated GTP-binding protein 1 OS=Homo sapiens OX=9606 GN=DRG1 PE=1 SV=1 | 40.5422 | 367 |
| Q9Y490 | TLN1_HUMAN | TLN1 | Talin-1 OS=Homo sapiens OX=9606 GN=TLN1 PE=1 SV=3 | 269.7671 | 2541 |
| Q9Y4L1 | HYOU1_HUMAN | HYOU1 | Hypoxia up-regulated protein 1 OS=Homo sapiens OX=9606 GN=HYOU1 PE=1 SV=1 | 111.3354 | 999 |
| Q9Y5C1 | ANGL3_HUMAN | ANGPTL3 | Angiopoietin-related protein 3 OS=Homo sapiens OX=9606 GN=ANGPTL3 PE=1 SV=1 | 53.6375 | 460 |
| Q9Y5Y7 | LYVE1_HUMAN | LYVE1 | Lymphatic vessel endothelial hyaluronic acid receptor 1 OS=Homo sapiens OX=9606 GN=LYVE1 PE=1 SV=2 | 35.2134 | 322 |
| Q9Y6R7 | FCGBP_HUMAN | FCGBP | IgGFc-binding protein OS=Homo sapiens OX=9606 GN=FCGBP PE=1 SV=3 | 572.0167 | 5405 |
| Q9Y6Z7 | COL10_HUMAN | COLEC10 | Collectin-10 OS=Homo sapiens OX=9606 GN=COLEC10 PE=1 SV=2 | 30.705 | 277 |

# *Table S2 Differentially expressed proteins in breast cancer and healthy group were quantified*

| **Proteins** | **Protein Names** | **Genes** | **Fold change** | **P value** |
| --- | --- | --- | --- | --- |
| A0A075B6K4 | LV310_HUMAN | IGLV3-10 | 0.506502446 | 0.026852551 |
| P15259 | PGAM2_HUMAN | PGAM2 | 0.336088918 | 0.000182672 |
| P22314 | UBA1_HUMAN | UBA1 | 0.489282962 | 0.019972989 |
| A0A0C4DH35 | HV335_HUMAN | IGHV3-35 | 0.676946661 | 0.019421397 |
| P23528 | COF1_HUMAN | CFL1 | 0.694207428 | 0.001840375 |
| P27797 | CALR_HUMAN | CALR | 0.654698187 | 0.027780569 |
| P27918 | PROP_HUMAN | CFP | 0.648793431 | 0.008671185 |
| P31943 | HNRH1_HUMAN | HNRNPH1 | 0.296911871 | 5.92194E-05 |
| P34932 | HSP74_HUMAN | HSPA4 | 0.560056105 | 0.019010751 |
| P41250 | GARS_HUMAN | GARS1 | 0.328330749 | 0.000658805 |
| P43243 | MATR3_HUMAN | MATR3 | 0.375651568 | 0.000155283 |
| P55209 | NP1L1_HUMAN | NAP1L1 | 0.528802681 | 0.029125863 |
| P62258 | 1433E_HUMAN | YWHAE | 0.711374952 | 0.018341446 |
| P62829 | RL23_HUMAN | RPL23 | 0.379745506 | 0.003512944 |
| Q02878 | RL6_HUMAN | RPL6 | 0.491797595 | 0.00333232 |
| Q14974 | IMB1_HUMAN | KPNB1 | 0.711408589 | 0.035865959 |
| Q15166 | PON3_HUMAN | PON3 | 0.827474282 | 0.027250951 |
| Q15366 | PCBP2_HUMAN | PCBP2 | 0.404213935 | 0.003621466 |
| Q8IUX8 | EGFL6_HUMAN | EGFL6 | 0.740717883 | 0.031209013 |
| Q96PD5 | PGRP2_HUMAN | PGLYRP2 | 0.771629462 | 0.013117101 |
| Q9HDC9 | APMAP_HUMAN | APMAP | 0.77657094 | 0.032472407 |
| P21333 | FLNA_HUMAN | FLNA | 0.520045712 | 0.022179034 |
| P23083 | HV102_HUMAN | IGHV1-2 | 0.372034223 | 0.031209013 |
| Q9Y5C1 | ANGL3_HUMAN | ANGPTL3 | 0.501611 | 0.037635 |
| P04439 | HLAA_HUMAN | HLA-A | 0.328922 | 0.045155 |
| P13639 | EF2_HUMAN | EEF2 | 0.521881 | 0.002024 |
| O43707 | ACTN4_HUMAN | ACTN4 | 0.527307 | 0.000335 |
| P02730 | B3AT_HUMAN | SLC4A1 | 0.370505 | 0.00152 |
| P02760 | AMBP_HUMAN | AMBP | 0.773146 | 0.028068 |
| O00571 | DDX3X_HUMAN | DDX3X | 0.675651 | 0.0373 |
| P00736 | C1R_HUMAN | C1R | 0.769934 | 0.022358 |
| P04908 | H2A1B_HUMAN | H2AC4 | 0.699124 | 0.01133 |
| P05386 | RLA1_HUMAN | RPLP1 | 0.415006 | 0.016167 |
| P06744 | G6PI_HUMAN | GPI | 0.389857 | 0.003231 |
| P08603 | CFAH_HUMAN | CFH | 0.745417 | 7.29E-05 |
| P0DME0 | SETLP_HUMAN | SETSIP | 0.512231 | 0.003444 |
| A0A0C4DH36 | HV338_HUMAN | IGHV3-38 | 1.384785 | 0.048357 |
| Q15063 | POSTN_HUMAN | POSTN | 1.327773 | 0.03888 |
| Q4G0P3 | HYDIN_HUMAN | HYDIN | 1.903645 | 0.011066 |
| O95477 | ABCA1_HUMAN | ABCA1 | 1.754817 | 0.005919 |
| Q9H8L6 | MMRN2_HUMAN | MMRN2 | 1.267081 | 0.021471 |
| P13645 | K1C10_HUMAN | KRT10 | 3.396146 | 0.040052 |
| P01008 | ANT3_HUMAN | SERPINC1 | 1.234885 | 0.011744 |
| Q13790 | APOF_HUMAN | APOF | 1.221982 | 0.014145 |
| P01701 | LV151_HUMAN | IGLV1-51 | 1.997949 | 0.016442 |
| P68371 | TBB4B_HUMAN | TUBB4B | 1.735124 | 0.003741 |
| P02652 | APOA2_HUMAN | APOA2 | 1.324266 | 0.025748 |
| P04264 | K2C1_HUMAN | KRT1 | 4.459995 | 0.024654 |
| P35527 | K1C9_HUMAN | KRT9 | 5.063647 | 0.034493 |
| P35030 | TRY3_HUMAN | PRSS3 | 6.032531 | 0.021134 |
| P01706 | LV211_HUMAN | IGLV2-11 | 5.118481 | 0.045155 |
| P35030 | TRY3_HUMAN | PRSS3 | 6.032531 | 0.021134 |

# *Table S3 The top 5 enriched GO items (biological process, Cellular Component, Molecular Function)、DO items and KEGG items at breast cancer and non-breast cancer*

| **ONTOLOGY** | **ID** | **Description** | **Gene Ratio** | **Bg Ratio** | **P value** | **Genes** |
| --- | --- | --- | --- | --- | --- | --- |
| BP | GO:0009056 | catabolic process | 7/30 | 53/509 | 0.027156 | APOA2/GPI/PGAM2/PRTN3/PON3/PGLYRP2/ANGPTL3 |
| BP | GO:0006066 | alcohol metabolic process | 4/30 | 21/509 | 0.029079 | ABCA1/APOA2/APOF/ANGPTL3 |
| MF | GO:0016787 | hydrolase activity | 4/23 | 15/430 | 0.005766 | C1R/PGAM2/PRTN3/PRSS3 |
| MF | GO:0043178 | alcohol binding | 3/23 | 9/430 | 0.009125 | ABCA1/APOA2/APOF |
| MF | GO:0005496 | steroid binding | 3/23 | 10/430 | 0.01258 | ABCA1/APOA2/APOF |
| MF | GO:0052689 | carboxylic ester hydrolase activity | 2/23 | 5/430 | 0.024832 | PON3/APMAP |
| MF | GO:0008233 | peptidase activity | 4/23 | 25/430 | 0.037106 | C1R/PRTN3/PRSS3/HABP2 |
| MF | GO:0005215 | transporter activity | 3/23 | 16/430 | 0.047442 | ABCA1/APOA2/APOF |
| KEGG | hsa04979 | Cholesterol metabolism | 3/14 | 16/288 | 0.035257 | ANGPTL3/APOA2/ABCA1 |
| KEGG | hsa02010 | ABC transporters | 1/14 | 1/288 | 0.048611 | ABCA1 |
| KEGG | hsa00520 | Amino sugar and nucleotide sugar metabolism | 1/14 | 1/288 | 0.048611 | GPI |
| KEGG | hsa01250 | Biosynthesis of nucleotide sugars | 1/14 | 1/288 | 0.048611 | GPI |
| DO | DOID:303 | substance-related disorder | 3/19 | 16/383 | 0.038506 | 3075/3308/27329(Genes ID) |

GO, Gene Ontology; KEGG, Kyoto Encyclopedia of Genes and Genomes; BP, biological process; CC, Cellular Component; MF, Molecular Function

# *Table S4 The protein-protein interaction network based on the String database at breast cancer and non- breast cancer*

| **Accession1** | **Accession2** | **gene1** | **gene2** | **degree1** | **degree2** | **experimental** | **database** | **text mining** | **combined score** |
| --- | --- | --- | --- | --- | --- | --- | --- | --- | --- |
| P24158 | P27918 | PRTN3 | CFP | 1 | 3 | 297 | 0 | 284 | 0.636 |
| P27918 | P00736 | CFP | C1R | 3 | 2 | 0 | 0 | 710 | 0.734 |
| P27918 | P08603 | CFP | CFH | 3 | 3 | 0 | 0 | 725 | 0.725 |
| O43707 | P68371 | ACTN4 | TUBB4B | 2 | 2 | 0 | 0 | 275 | 0.431 |
| O43707 | P34932 | ACTN4 | HSPA4 | 2 | 5 | 0 | 0 | 244 | 0.428 |
| Q15166 | P02652 | PON3 | APOA2 | 2 | 3 | 0 | 0 | 460 | 0.474 |
| Q15166 | Q13790 | PON3 | APOF | 2 | 3 | 0 | 0 | 390 | 0.415 |
| P15259 | P06744 | PGAM2 | GPI | 1 | 2 | 0 | 800 | 261 | 0.953 |
| P34932 | P68371 | HSPA4 | TUBB4B | 5 | 2 | 0 | 0 | 166 | 0.46 |
| P34932 | P06744 | HSPA4 | GPI | 5 | 2 | 0 | 0 | 315 | 0.444 |
| P34932 | P31943 | HSPA4 | HNRNPH1 | 5 | 2 | 0 | 0 | 218 | 0.43 |
| P34932 | P62829 | HSPA4 | RPL23 | 5 | 1 | 0 | 0 | 108 | 0.401 |
| P31943 | P43243 | HNRNPH1 | MATR3 | 2 | 1 | 678 | 0 | 419 | 0.991 |
| P08603 | P00736 | CFH | C1R | 3 | 2 | 0 | 0 | 603 | 0.679 |
| P08603 | O95477 | CFH | ABCA1 | 3 | 3 | 0 | 0 | 425 | 0.452 |
| P02652 | Q13790 | APOA2 | APOF | 3 | 3 | 270 | 540 | 596 | 0.876 |
| P02652 | O95477 | APOA2 | ABCA1 | 3 | 3 | 0 | 0 | 742 | 0.746 |
| Q9Y5C1 | O95477 | ANGPTL3 | ABCA1 | 2 | 3 | 0 | 0 | 519 | 0.55 |
